# Elephant gestural communication during agonistic interactions: repertoire, intentionality, and goals

**DOI:** 10.1007/s10071-026-02108-7

**Published:** 2026-09-25

**Authors:** Vesta Eleuteri, Anna Letrari, Lucy Bates, Catherine Hobaiter, Angela Stoeger

**Affiliations:** 1 Department of Behavioral & Cognitive Biology, University of Vienna, Vienna, Austria; 2 Department of Psychology, University of Portsmouth, Portsmouth, UK; 3 School of Psychology and Neuroscience, University of St Andrews, St Andrews, UK; 4 Zoo Conservation Science Lab, Vienna Zoo, Vienna, Austria

**Keywords:** Elephant, Gestural communication, Intentionality, Language, Agonistic behaviour

## Abstract

Goal-directed intentionality is the ability to communicate with the intention of eliciting reactions from recipients. Long considered uniquely human, today it has been reported in the signalling of several species, most notably in the large, flexible gestural repertoires of non-human apes. Semi-captive elephants have also shown goal-directed intentionality when gesturing to humans. However, whether wild elephants gesture intentionally to conspecifics has not been systematically explored. Elephants frequently engage in agonistic interactions, in which goal-directed intentional communication may help resolve conflict and prevent escalation. Here, we systematically investigated intentional gesturing in wild African savannah elephants by examining agonistic interactions in the Addo and Marakele populations in South Africa. Elephants used 41 gesture types (derived from 32 gesture actions) to communicate two main goals: “Move away” and “Stop behaviour”. The gesture types used by the populations largely overlapped, suggesting a shared context-specific repertoire for agonistic interactions. However, the populations differed in the gesture types they used most frequently and in the resources they competed over (water in Addo, reeds in Marakele). Our study supports the convergent evolution of extensive and flexible intentional gestural communication in elephants, providing insights into the drivers of elephant agonistic behaviour and highlighting potential conservation implications.

**Supplementary Information:**

The online version contains supplementary material available at https://doi.org/10.1007/s10071-026-02108-7.

## Introduction

Studies on elephant communication have traditionally focused on their acoustic and chemical signals, likely due to the species’ acute hearing and sense of smell (Stoeger 2021; Schulte and LaDue 2021; Poole and Granli 2021). However, elephants also use conspicuous visual, tactile, and multisensory signals in different contexts, from affiliation and greeting to play and sexual advertisement, suggesting vision and touch are also key for communication (Langbauer 2000; Kahl and Armstrong 2002; Poole and Granli 2011, 2021; Jacobson and Plotnik 2020). For example, in African savannah elephants (*Loxodonta africana*), individuals affiliate or greet by touching each other with their trunks (Poole and Granli 2011; Allen et al. 2021; Eleuteri et al. 2024), while males signal musth—a male-specific sexual hormonal state—by waving their ears and curling their trunks (Poole and Moss 1981; Poole 1987, 1989). While there are rich descriptions of elephant signal repertoires (see Eleuteri et al. 2026 for a review), previous studies have not yet determined whether elephants use their signals intentionally to achieve specific goals.

Intentionality is a defining feature of human language. It represents the ability to communicate underlying thoughts to specific recipients in order to influence their behaviour and/or understanding (Grice 1957, 1969; Tomasello 2010). Intentionality has been operationalised as hierarchical orders of increasing cognitive complexity. First-order, or goal-directed, intentionality refers to communicating the intention (or goal) of eliciting a specific reaction in the recipient (e.g., asking for help; Dennett 1983, 1989). Second-order intentionality implies the intention of changing the recipient’s mental states (e.g., informing someone that one is hurt), while higher orders involve recursive mental-state attribution between the signaller and recipient (e.g., wanting you to know that I know that you know I am hurt). To date, there is no conclusive evidence that animals signal with the intention of changing others’ mental states, although some studies suggest signalling that takes recipients’ mental states into account (Cartmill and Byrne 2007; Crockford et al. 2012). In contrast, first-order, goal-directed, intentionality has been documented in the signalling of several taxa (Aychet et al. 2025).

Because others’ minds are not directly accessible to observers, comparative researchers studying goal-directed intentional communication (hereafter, intentional communication) have relied on behavioural indicators derived from studies of pre-linguistic infants, enabling cross-species comparisons (Bates et al. 1975; Tomasello et al. 1994; Leavens et al. 2005; Townsend et al. 2017; Ben Mocha and Burkart 2021). First, signallers should exhibit behaviours indicating that signals are directed to an audience (here, *audience directedness*) by a): signalling in the presence of a potential recipient (social use); b) visually checking the recipient and their state of visual attention (audience checking); and c) adjusting signal sensory modality according to the recipient’s attentional state, for example by using tactile or audible signals when recipients are not visually attentive (sensitivity to recipient attention). Second, signallers should show evidence of communicating in pursuit of a goal (here, *goal directedness*) by d): pausing after signalling to allow the recipient the opportunity to react in a way that meets the signaller’s goal (response waiting); and e) continuing to signal with the same or different signal types if the goal is not achieved (persistence, including elaboration). Using these criteria, a signal’s goal can be inferred from a recipient behavioural reaction that i) plausibly reflects the signaller’s intention (e.g., excluding aggressive reactions) and ii) appears to satisfy the signaller, as indicated by their cessation of signalling once the reaction occurs (Cartmill and Byrne 2010; Hobaiter and Byrne 2014).

While intentionality has been reported in specific chimpanzee vocalizations (Crockford et al. 2012, 2015; Gruber and Zuberbühler 2013; Schel et al. 2013; Bouchard and Zuberbühler 2022), most evidence comes from animal gestures (Rodrigues et al. 2021; Aychet et al. 2025). In primatology, gestures are typically defined as intentionally produced physical movements of different body parts—including limbs, head, torso, face, and even whole body—that are mechanically ineffective at achieving a goal (Hobaiter and Byrne 2011a; Gupta and Sinha 2019; Molesti et al. 2020). Outside primate species, intentionality has been typically shown in the use of a limited set of gesture types associated with a particular goal (Xitco et al. 2001; Pika and Bugnyar 2011; Vail et al. 2013; Ben Mocha and Pika 2019; Ben Mocha et al. 2019; but see studies on dog–human interactions: Savalli et al. 2014; Worsley and O’Hara 2018). In primates, intentional gesturing is more widespread, with several haplorrhine primates using repertoires of dozens of gesture types (c.f.,: Aychet et al. 2025; macaques: Gupta and Sinha 2019; baboons: Molesti et al. 2020; mangabeys: Schel et al. 2022; spider monkeys: Villa-Larenas et al. 2024). Among primates, non-human ape gesture has been most extensively studied (Byrne et al. 2017; Tomasello 2019). All apes communicate intentionally a wide range of goals with repertoires of up to 90 core gesture actions expressed into more than 150 gesture types according to different production features (e.g., the body part used, the recipient body part touched, the presence of repetition; c.f., Mielke et al. 2024; bonobos: Pika et al. 2005; Graham et al. 2017, 2018; chimpanzees: Tomasello et al. 1985; Liebal et al. 2004; Hobaiter and Byrne 2011a, 2014; Roberts et al. 2014; Mielke et al. 2024; mountain gorillas: Grund et al. 2025; western gorillas: Pika et al. 2003; Genty et al. 2009; orangutans: Bard 1992; Liebal et al. 2006; Cartmill and Byrne 2010; Knox et al. 2019). Moreover, apes gesture flexibly across goals, employing the same gesture to communicate different goals and different gestures to communicate the same goal (Cartmill and Byrne 2010; Hobaiter and Byrne 2014; Graham et al. 2018).

Although some primate species possess flexible intentional gestural repertoires (c.f., Byrne et al. 2017; Tomasello 2019; Aychet et al. 2025), it remains unclear whether such systems reflect differences in their cognitive or communicative abilities, or a bias in research effort (Aychet et al. 2025). One influential hypothesis proposes that social complexity promotes the evolution of cognitive—and by extension, communicative—complexity (Dunbar 1998; Byrne and Bates 2007; Freeberg et al. 2012). Specifically, living in large and complex social systems may impose communicative demands that favour the evolution of extensive and flexible signal repertoires (Freeberg et al. 2012; Peckre et al. 2019). Some primate species live within elaborate social systems involving diverse relationships, including with kin, allies, and competitors (Wrangham 1979; Pougnault et al. 2022; Graham et al. 2022; Badihi et al. 2022), potentially favouring the need of large, flexible, intentional gestural repertoires. However, comparisons within the primate order are challenging, as it is difficult to disentangle what is shaped by phylogenetic proximity and what may be due to similar selective social pressures. Examining species with similarly rich complex social lives but that are phylogenetically distant is therefore important for assessing the extent to which social complexity has favoured the evolution of extensive and flexible intentional gestural communication systems beyond the (haplorrhine) primate lineage.

African savannah elephants (hereafter, elephants) last shared a common ancestor with primates more than 100 million years ago (Hedges 2001; Murphy et al. 2001). In addition, whereas primates rely primarily on vision and exhibit refined manual dexterity, elephants depend predominantly on hearing and olfaction and use their highly flexible, multifunctional trunk for prehension and manipulation (Byrne et al. 2009; Jacobson and Plotnik 2020; Bates 2020). Despite these differences, elephants, like some primates, are large-brained, long-lived animals, and live in multi-level fission–fusion social systems (Douglas-Hamilton 1972; Wittemyer et al. 2005; Evans and Harris 2008; Archie et al. 2011). In females, the first social level consists of the mother–offspring unit, which typically associates with related females and their offspring in family or core groups led by an older, more experienced matriarch (Douglas-Hamilton 1972; Wittemyer et al. 2005; Archie et al. 2011). Family groups that regularly associate form bond groups, usually composed of closely related individuals, while multiple family groups sharing the same dry season range are considered clans (Wittemyer et al. 2005, 2009; Archie et al. 2005). Male elephants live in a more fluid social system: after adolescence, males gradually leave their natal family groups and may roam alone or with family groups, but frequently join bachelor groups with other males (Lee et al. 2011; Morris-Drake and Mumby 2018). Elephants may thus benefit from an extensive and flexible intentional gestural system to mediate diverse social interactions and relationships. Recent studies have shown that semi-captive African savannah and Asian elephants exhibit audience directedness when gesturing to both humans and conspecifics (Smet and Byrne 2014; Eleuteri et al. 2024; Jim et al. 2025), and goal directedness when gesturing to humans (Eleuteri et al. 2025). However, whether wild elephants gesture intentionally to conspecifics has not been systematically investigated.

Agonistic interactions provide a promising context for a first investigation of goal-directed intentional communication in wild elephants. Many species engage in agonistic interactions when competing over resources, confronting threats, or asserting dominance, and these interactions can escalate into costly physical aggression, risking injury or even death (Archer 1988; Huntingford 2013; Wilson et al. 2014). Under such competitive conditions, signalling can provide a lower-cost alternative to direct physical confrontation (Smith and Price 1973; Paton and Caryl 1986; Tibbetts 2013; Pellis and Pellis 2015). In particular, communicating goals—such requesting that a recipient moves away from a resource—may allow individuals to resolve competition while avoiding direct physical conflict. Several species engage in agonistic signalling: for example, male red deer roar when competing over mates (Clutton-Brock et al. 1982; Clutton-Brock and Huchard 2013), while several birds use vocal or visual displays during territorial disputes (Deag and Scott 1999; Kenyon and Martin 2022). Although it remains largely unknown whether agonistic signalling is intentional in most species, during agonistic interactions primates use intentionally a large number of gesture types leading to clear recipient reactions that satisfy the signaller’s apparent goal (e.g., requesting the recipient to move away or stop their behaviour; Hobaiter 2011; Hobaiter and Byrne 2014; Molesti et al. 2020). Elephants also use a range of physical movements during agonistic interactions (Freeman et al. 2004; Archie et al. 2006; Poole and Granli 2011, 2021; O’Connell-Rodwell et al. 2011). They may strike competitors with their trunks, fling their trunks toward them, or spread their ears forward, likely to appear larger (Kahl and Armstrong 2000; Poole and Granli 2011, 2021). However, whether these movements represent intentionally produced gestures has not been investigated.

Although elephants are endangered at the continental level (Gobush et al. 2022), in South Africa their confinement to fenced parks or reserves restrict their natural movements and can thus lead to high population densities (Van et al. 2006; Pretorius et al. 2019). Such conditions may increase competition over available resources and, consequently, result in a higher risk of escalation. Exploring intentional communication during agonistic interactions in fenced populations is thus particularly important, as it can shed light on the drivers of competition in restricted environments and thereby inform park management strategies to mitigate unsustainable aggression (Balfour et al. 2007).

In this study, we systematically investigated gestural communication during agonistic interactions in wild elephants by focusing on two fenced, highly dense populations living in the Marakele and Addo Elephant National Parks (Van Staden 2002; Whitehouse and Kerley 2002; Reardon 2021). Our primary aim was to test whether wild elephants in these populations gesture intentionally during agonistic interactions and to provide a systematic description of the gesture forms used and their associated goals in this context. There is substantial variation in both which and how many criteria have been used to establish intentionality across species (see Townsend et al. 2017; Graham et al. 2020; Rodrigues et al. 2021; Rodrigues and Fröhlich 2021), and no single criterion can conclusively rule out simpler alternatives (e.g., arousal, Dennett 1983; Ben Mocha and Burkart 2021; Rodrigues and Fröhlich 2021). A robust approach therefore requires assessing a suite of intentionality criteria (c.f., Townsend et al. 2017; Ben Mocha and Burkart 2021). Here, we tested elephant gestural communication against all key criteria of intentionality: audience checking, sensitivity to recipient attention, response waiting, and persistence. Based on evidence from semi-captive individuals (Eleuteri et al. 2024, 2025), we predicted that wild elephants would show audience checking before and during gesturing, as well as sensitivity to recipient attention by selecting gesture sensory modalities that recipients could perceive (e.g., tactile or acoustic when recipients are not visually attentive). We further predicted that, after gesturing, elephants would exhibit response waiting and persistence if recipients did not react in ways that fulfilled their apparent goal (Eleuteri et al. 2025). We last expected that elephants—similarly to apes (Hobaiter 2011; Byrne et al. 2017)—would possess a large repertoire of intentional gestures used flexibly across goals during agonistic interactions.

We also examined potential population-level differences in the gestures and goals associated with agonistic interactions. Based on evidence that apes largely share gestural repertoires across populations and even species (reviewed in Byrne 2017), we predicted that Addo and Marakele elephants would similarly largely share gesture types during agonistic interactions. However, because elephants are believed to rely heavily on socially mediated learning (Fishlock et al. 2016; Bates et al. 2025), we expected the populations to differ in their “repertoires of regular use”, namely the subset of gesture types used most frequently (Badihi 2024). In addition, we investigated whether the two populations differed in their success at achieving goals and in rates of gestural persistence. Elephants often compete over limited and concentrated resources such as waterholes (Archie et al. 2006; Wittemyer and Getz 2007), particularly during the dry season (Ferry et al. 2016). In Marakele, water is abundant year-round, due to a perennial river flowing through the park (Van Staden 2002; Van Staden et al. 2020). In contrast, Addo lacks natural perennial water sources, and during the dry season animals rely primarily on artificial waterholes distributed throughout the park (Brett 2019; Reardon 2021). Because water is more limited and spatially concentrated in Addo, it is likely a source of greater competition, potentially favouring the use of more effective gesture types and/or higher rates of persistence. By systematically examining the intentional use, forms, and goals of gestures during agonistic interactions in two populations, this study provides a foundation for understanding intentional gestural communication in wild elephants.

## Methods

### Study sites and subjects

#### Marakele national park

The Marakele National Park (Marakele) is a 67,000 ha fenced park located in the Limpopo Province of South Africa (Van Staden et al. 2020). The park lies on the extreme south-western quadrant of the Waterberg Mountain Range and is one of the youngest national parks in South Africa. In 1988 it was declared as Kransberg National Park and has been expanded over the years by including nearby private farms (Van Staden 2002). The founding elephant population consisted of 39 individuals first introduced in 1996 (Van Staden 2002). Today, the elephant population consists of an estimated 400 elephants (SAN Parks 2024), of which around 80 adult males and around 60 adult females are individually identified (within ~15 family groups).

### Addo elephant national park

The Addo Elephant National Park (Addo) is a 162,000 ha fenced park located in the Eastern Cape Province of South Africa. The elephants roam in a sub-fenced area of 88,000 ha (park management, personal communication). The park was founded in 1931 to safeguard eleven individuals who had survived historical poaching and hunting (Whitehouse and Hall-Martin 2000; Reardon 2021). Today there are ~700 elephants from an estimated 25 family groups descended from six matrilines (Whitehouse and Harley 2001). Currently, around 200 adult males are individually identified, while most adult females are not identified, as female identification is particularly challenging in this population due to their widespread absence of tusks (Reardon 2021).

## Data collection

In Marakele, data collection took place in the Marataba Contractual National Park private section between October and December 2022 and in September 2024, and in the Addo Elephant National Park (South Africa) between November and December 2023. Data were collected during daylight hours (6 am-6 pm in Marakele; 8 am-5 pm in Addo). Daily data collection was longer in Marakele due to the challenge of finding elephants in large aggregations. In Marakele, we focused our observations along the river of the Marataba section, while in Addo data were recorded at the main water holes.

Data consisted of videos collected with a Panasonic HC-VFX1 video-camera using an all-occurrence approach (Altmann 1974). When elephants were encountered, VE directed the video-camera towards the elephants using the pre-record function, which continuously captures and deletes footage of up to 4 s before the record button is pressed, and thus reduces the likelihood that the start of an interaction of interest is missed. We started a recording whenever an interaction with potential for gestural communication began or was anticipated and ended the recording when the interaction ended or after subsequent interactions ended. Despite the use of pre-record function, it is possible that in some cases recordings started after an interaction had already begun. In total we collected ~59 h of video from Marakele and ~32 h from Addo.

## Data pre-processing

We collected 2469 videos of elephant interactions (1476 from Marakele and 993 from Addo). Videos were then transferred to a MacBook Pro and meta-data (e.g., date, time, the interactional context(s), and the presence of codable gestures) was entered into a FileMaker database. Agonistic interactions consisting of elephants competing over a resource (e.g., water or food), confronting threats (e.g., an adult male approaching a family group), or displacing others (Wittemyer and Getz 2007; O’Connell-Rodwell et al. 2011) were extracted along with videos of aggression (i.e., forcefully attacking the recipient). In Addo, 321/993 videos (32.3%) contained agonistic interactions and 27 (2.7%) physical aggression; in Marakele, 101/1476 videos (6.8%) included agonistic interactions and 2 (0.1%) physical aggression. Of these, a subset of 127 videos included codable gestures (79 from Addo and 48 from Marakele) and were thus selected for video coding.

## Definitions

In ape studies, intentionality is well-established and the definition of gesture typically requires evidence of intentional use (Byrne et al. 2017). As our goal was to investigate the presence of intentional gesturing in elephants, we first considered all potential gestures in order to be able to test for their intentional use. A *gesture* was thus defined as any mechanically ineffective physical movement used in a social situation (i.e., when the elephant was not alone and therefore a potential recipient was present; following Eleuteri et al. 2025). Mechanically effective movements—such as physically pushing a recipient to fully displace them—as well as locomotion, feeding, or self-directed activities, were excluded (following Genty et al. 2009; Hobaiter and Byrne 2011a).

Component-based approaches can help reduce coding bias towards pre-defined signal categories, allow for finer granularity in signal coding and analysis, and ensure that coded units are consistently defined, facilitating replicability across populations or species (Parr and Waller 2006; Vick et al. 2007; Waller et al. 2020; Grund et al. 2023; Mielke et al. 2024). Thus, to identify gesture forms, we adopted the GesturalOrigins component-based approach (Grund et al. 2023; Mielke et al. 2024; Eleuteri et al. 2024). Here we incorporated gesture actions and signaller body parts in defining gesture types. We first defined the *gesture action* as the basic physical movement that uniquely characterizes a gesture form (e.g., Fling, Swing, Shake; following, Grund et al. 2023), and then defined the *gesture type* as the combination of a gesture action and the body part used to produce it (e.g., Trunk Fling, Head Fling; c.f., also termed gesture morphs or units, Mielke et al. 2024). To establish the initial ethogram of gesture actions, we reviewed the ElephantVoices ethogram, a comprehensive database of over 322 behaviours across 23 contexts derived from observations of three elephant populations: Amboseli and Maasai Mara in Kenya and Gorongosa in Mozambique (Poole and Granli 2021). Where we identified repeated instances of movements that did not match any of the database’s descriptions, we added new gesture actions. We then classified gesture actions according to their sensory modality: *silent-visual* comprised only visual information (e.g., Reach); *tactile* involved non-accidental physical contact with the recipient (e.g., Hit recipient); and *audible* produced an audible sound as an intrinsic part of the gesture action (e.g., Flap; following Hobaiter and Byrne 2011a; Eleuteri et al. 2024).

We further defined a *gesture token* as a single instance of gesture use. For example, a Trunk Fling followed by an Ear Flap would represent two gesture tokens**.** A *gestural communication event* included all gesture tokens produced from a signaller to a recipient on a particular occasion/interaction, and could comprise a single gesture token, or multiple tokens produced in series. Following previous studies that established a one-second baseline to distinguish response waiting and identify persistence within serial gesturing (Genty and Byrne 2010; Hobaiter and Byrne 2011b), we defined series of gesture tokens within a gestural communication event as either *sequences*—gesture tokens separated by less than one second; or *bouts*—gesture tokens and/or sequences separated by more than one second (See Sequence part and Bout part in Table S1for information on how these variables were coded). We then defined the apparent *goal* of the gestural communication event as the recipient reaction that apparently satisfied the signaller, as indicated by their cessation of gesturing (also termed Apparently Satisfactory Outcome; Cartmill and Byrne 2010 and Hobaiter and Byrne 2014). Using this approach, all gesture tokens in the gestural communication event were ascribed the same goal. As in apes, we initially defined two main goal categories during agonistic interactions (Cartmill and Byrne 2010; Hobaiter and Byrne 2014; Graham et al. 2018): “Move away” for when the recipient moved away from the signaller or a location, and “Stop behaviour” for when the recipient stopped any behaviour in which they were previously engaged. When the recipient’s reaction involved competition over food or water, we subsequently specified the type of resource under specific goal categories (e.g., “Move away from water/reeds”, “Stop behaviour at water/reeds”).

As we collected videos from social situations, all gestural communication events inherently fulfilled the intentionality criterion of social use (i.e., gesturing in the presence of a potential recipient; Tomasello et al. 1985; Townsend et al. 2017). We thus established whether elephants gestured intentionally by fulfilling other key criteria: audience checking, sensitivity to recipient attention, response waiting, and persistence (Table 1).

**Table 1:** Definitions of the criteria used to test for goal-directed intentionality during elephant agonistic interactions

Intentionality criteria	Definitions
Audience checking	The signaller visually attends to the recipient at the *onset* and/or *during* the production of gesture token(s)* (See Table S1 for information on coding of Gaze before and Gaze during)
Sensitivity to recipient attention	The signaller shows sensitivity to the visual attentional state of the recipient when producing the gesture token. Specifically, the signaller selects tactile or audible gesture actions, as compared to silent-visual ones, when the recipient is not visually attending to them (e.g., signaller is not in the line of sight of the recipient, see Recipient visual attention in TableS1)
Response waiting	After producing gesture token(s)*, the signaller pauses for at least one second while visually attending to the recipient and without engaging in other activities
Persistence	After response waiting, the signaller produces further gesture token(s)

* We use “gesture token(s)” in the case of gesture sequences (see definition above), for which intentionality criteria are typically assessed at the level of the sequence (rather than the individual comprised gesture tokens) because gestures, unlike vocalisations, can also be produced while overlapping (Hobaiter and Byrne 2011a; Byrne et al. 2017)

## Video coding

We coded videos using the video coding software Elan 6.2. Our coding method consisted of an elephant-adapted version of the GesturalOrigins coding framework (See Table S1 for full video coding scheme and the GesturalOrigins framework resources: Grund et al. 2023; https://github.com/CharlotteGrund/Gestural_Origins_Coding-methods_paper). Videos were coded at three levels of detail: the gestural communication event (i.e., Communication number), the gesture record, and the bout part. The gestural communication event included information on the signaller and recipient identity, the signaller prior and post communication interactional context (Fröhlich et al. 2025), and the goal. The gesture record included information on the gesture token produced (e.g., gesture action, body part used). The bout part included information on the intentionality criteria, which were coded as nominal variables (e.g., Yes, No, Unk) (See Table S1 for information on all coding variables).

To establish the reliability of our video coding, we conducted inter-observer reliability on ~10% of gestural communication events randomly selected from Addo (original coder: AL, secondary coder: VE) and ~10% from Marakele (original coder: VE, secondary coder: AL). We first assessed whether the same number of gesture tokens were coded in the gestural communication event. Then, for the common gesture tokens coded, we assessed inter-observer reliability for the variables: Gesture record, Body part, Recipient visual attention, Bout part, Gaze before, Gaze during, Response waiting, Persistence, Goal, Goal met (Table S1). In both populations we found substantial to almost perfect reliability on all variables (See Table S2).

## Statistical analyses

### Intentionality

Because not all signallers could be individually identified, we employed a cautious approach by testing each intentionality criterion (Table 1) with two sets of analyses. In the first, we kept the full dataset including both identified and unidentified individual signallers. In the second, we adopted a more conservative approach by including only data from identified signallers (i.e., identified dataset), in which we could control for pseudoreplication where individuals contributed varying amounts of data. For the full dataset, we used binomial tests and chi-squared tests (Agresti 2012; McHugh 2013). For the identified dataset, we used generalised linear mixed models (binomial (logit) and multinomial (multilogit) GLMMs; Baayen 2008), including only signallers contributing at least four gesture tokens to allow our inclusion of Signaller as a random effect (to control for pseudoreplication). We assessed model stability by comparing the full model estimates with those from models from which random effect levels were removed one at a time, and checked for overdispersion (Nieuwenhuis et al. 2012).

*Audience checking:* We assessed audience checking both at the *onset* and *during* the bout part using binomial tests for the full dataset and corresponding logistic GLMMs for the identified dataset. In the GLMMs, the response variables were, respectively, “Gaze before” and “Gaze during” (coded as “Yes” when audience checking occurred and “No” when not). We excluded bout parts where audience checking was unknown or the signaller was out of sight (See Table S3 for original and trimmed sample size numbers). The final sample sizes were n=539 (binomial test) and n=324 (GLMM) for the *Gaze before* models, and n=546 (binomial test) and n=322 (GLMM) for the *Gaze during* models. Both GLMMs were stable (Table S4) and showed no overdispersion (Gaze before overdisp value=0.369, p=0.272; Gaze during overdisp value=0.425, p=0.392).

*Sensitivity to recipient attention:* We tested this criterion at the level of the gesture token (not bout part) since the sensory modality is specific to the gesture action produced (See Definitions). First, we assessed whether signallers produce more gesture tokens when recipients were visually attentive using a binomial test for the full dataset and a corresponding logistic GLMM for the identified dataset. In the GLMM, the response variable was Recipient visual attention (coded as “Yes” when they were visually attending and “No” when not). Gesture tokens where recipient visual attention was unknown or the recipient was out of sight were excluded (Table S3 for original and trimmed sample size numbers). The final sample sizes were n=810 (binomial test) and n=489 (GLMM). The GLMM was stable (Table S4) and showed no overdispersion (overdisp value=1.025, p=0.872).

Then we assessed whether signallers varied the sensory modality of the gesture according to the recipient’s visual state of attention. We used a chi-squared test for the full dataset and a corresponding multinomial logit model (mblogit) for the identified dataset (Agresti 2012). In the mblogit the response was Gesture modality (silent-visual, tactile, audible) and the predictor was Recipient visual attention (following Eleuteri et al. 2024). To test the overall effect of the predictor, we compared the full model with the reduced model excluding the predictor but including the random effect using a likelihood ratio test (Dobson and Barnett 2018). We excluded three gesture tokens from gesture actions where the sensory modality was unknown (i.e., Trunk Reach_Touch_Unc, where it was unclear if the extension of the Trunk made contact with the recipient (Touch) or not (Reach); See Table S3 for original and trimmed sample size numbers). The final sample sizes were n=807 (chi-squared test) and n=486 (mblogit). In addition, following previous studies (Hobaiter and Byrne 2011a; Eleuteri et al. 2024), we used a percentage-variation plot to examine whether elephants adapt their gesture sensory modality according to the recipient’s visual state of attention, relative to the overall use of that modality in the dataset. To do so, we first calculated the overall proportions of silent-visual, audible, and tactile gesture tokens in the dataset. Then, we calculated the proportions of use of each modality when recipients were visually attending and when they were not. Finally, to quantify modality adaptation, we computed the percentage deviation as: (β/α − 1) × 100, where β is the proportion of a given modality (e.g., tactile) in a given visual attention state (e.g., attending) and α is its overall proportion in the dataset. Positive deviations above zero indicate that signallers increase their use of that modality (relative to its baseline distribution) according to the attentional state of the recipient. We again made one plot for the full dataset (n tokens=807) and one for the identified dataset (n tokens=486).

*Response waiting*: We tested for response waiting after each bout part using a binomial test for the full dataset and a corresponding logistic GLMM for the identified dataset. In the GLMM the response variable was Response waiting (coded as “Yes” when there was response waiting and “No” when not). Bout parts where response waiting was “Unknown” were excluded (See Table S3 for original sample size numbers). The final sample sizes were n=531 (binomial test) and n=318 (GLMM) bout parts. The GLMM was stable (Table S4) and showed no overdispersion (overdisp value=0.816, p=0.640).

*Persistence:* We also tested for persistence after each bout part, again using a binomial test for the full dataset and a corresponding logistic GLMM for the identified dataset. In the GLMM, the response variable was Persistence (coded as “Yes” when there was persistence and “No” when not). We excluded bout parts where persistence was “Unknown” and removed the final bout part in gestural communication events where the goal was met, as signallers would not persist following a satisfactory recipient reaction (See Table S3 for original and trimmed sample size numbers). The final sample sizes were n=404 (binomial test) and n=242 (GLMM) bout parts. The GLMM was stable (Table S4) and showed no overdispersion (overdisp value=1.095, p=0.608).

### Agonistic intentional gestural repertoire and goals

To describe the gestural repertoire used intentionally by elephants during agonistic interactions, we excluded gesture types that never resulted in the recipient reacting in a way that satisfied the signaller’s goal (Hobaiter and Byrne 2011a; Townsend et al. 2017). To minimise observer error or over-coding, we further excluded gesture types with fewer than three gesture tokens observed (Hobaiter and Byrne 2011a; Graham et al. 2017; Grund et al. 2025). We also removed three gesture tokens in which the gesture action was unclear, as we could not assess whether the signaller reached towards or made contact with the recipient, leading to a final sample size of n=810 gesture tokens.

To investigate the goals of elephant gesture types in agonistic interactions, we included only successful gestural communication events, namely where the recipient reacted by meeting the signaller’s apparent goal (n events=168 including n=545 tokens; Schel et al. 2022). We then examined the main goals (i.e., Move away; Stop behaviour) of gesture types contributing at least three gesture tokens (n tokens=507). We considered gesture types where a single main goal was expressed in at least 70% of cases as being tightly associated with a primary main goal (Cartmill and Byrne 2010; Hobaiter and Byrne 2014).

In addition, to further specify the goals of elephant gestures, we first examined the recipient’s interactional context prior to each main goal, focusing on contexts with at least three successful gestural communication events and excluding events where the context was unknown (n events=131 including n=448 tokens). Then, we assessed whether gesture types were used specifically or flexibly to communicate more specific goals (i.e., Move away from water/reeds; Stop behaviour at water/reeds) within each recipient prior context, again excluding gesture types with fewer than three gesture tokens (n=417 tokens).

### Population differences in agonistic gesturing

For our population comparison of elephant gestural communication during agonistic interactions, we first assessed to what extent we had fully catalogued the intentional gesture actions and gesture types used by each population in this context. We did so by visually checking whether the cumulative number of both gesture actions and gesture types identified against the number of gesture tokens coded reached asymptote respectively within each population. We then assessed whether the two populations differed in the gesture types used and in their “repertoire of regular use”, defined as the subset of gesture types which, when ranked from highest to lowest frequency, cumulatively accounted for 70% of all gesture tokens within the population (Hobaiter 2011; Grund et al. 2025). Lastly, we explored whether the two populations differed in the resources over which they competed.

Finally, we tested whether the populations varied in the success of their gesturing at achieving goals and in rates of gestural persistence using two logistic GLMMs. In the Population Outcome GLMM, we included whether the goal was met at the end of the gestural communication event (i.e., Outcome, coded as “Reaction” when the goal was met and “No_reaction” when not; See Table S1). In the Population Persistence GLMM, the response variable was Persistence (coded as “Yes” when signallers showed persistence and “No” when not). In both models, Population was the predictor and Signaller the random effect (to control for pseudoreplication). To explore the overall effect of the predictor, we compared the full model with the reduced model without the predictor but including the random effect using a likelihood ratio test (Dobson and Barnett 2018). We again assessed model stability by comparing the full model estimates with those from models from which random effect levels were removed one at a time, and also assessed overdispersion (Nieuwenhuis et al. 2012). For the Population Outcome GLMM we removed bout parts where it was unclear whether the goal was met or where the gestural communication event ended due to external circumstances (e.g., the recording was interrupted), leading to a final sample size of n=327 bout parts. The sample size for the Population Persistence GLMM was again n=242 bout parts. Both GLMMs were stable (Table S4) and showed no overdispersion (Population Outcome overdisp value=1.019, p=0.984; Population Persistence overdisp value=1.090, p=0.616). All analyses above were conducted with R v. 4.0.2 using the packages *lme4* v 1.1–37 (Bates et al. 2015) and *mclogit* v 0.9.6 for GLMMs, and *performance* for overdispersion tests (Lüdecke et al. 2021).

## Results

### Intentionality

**Fig. 1:**
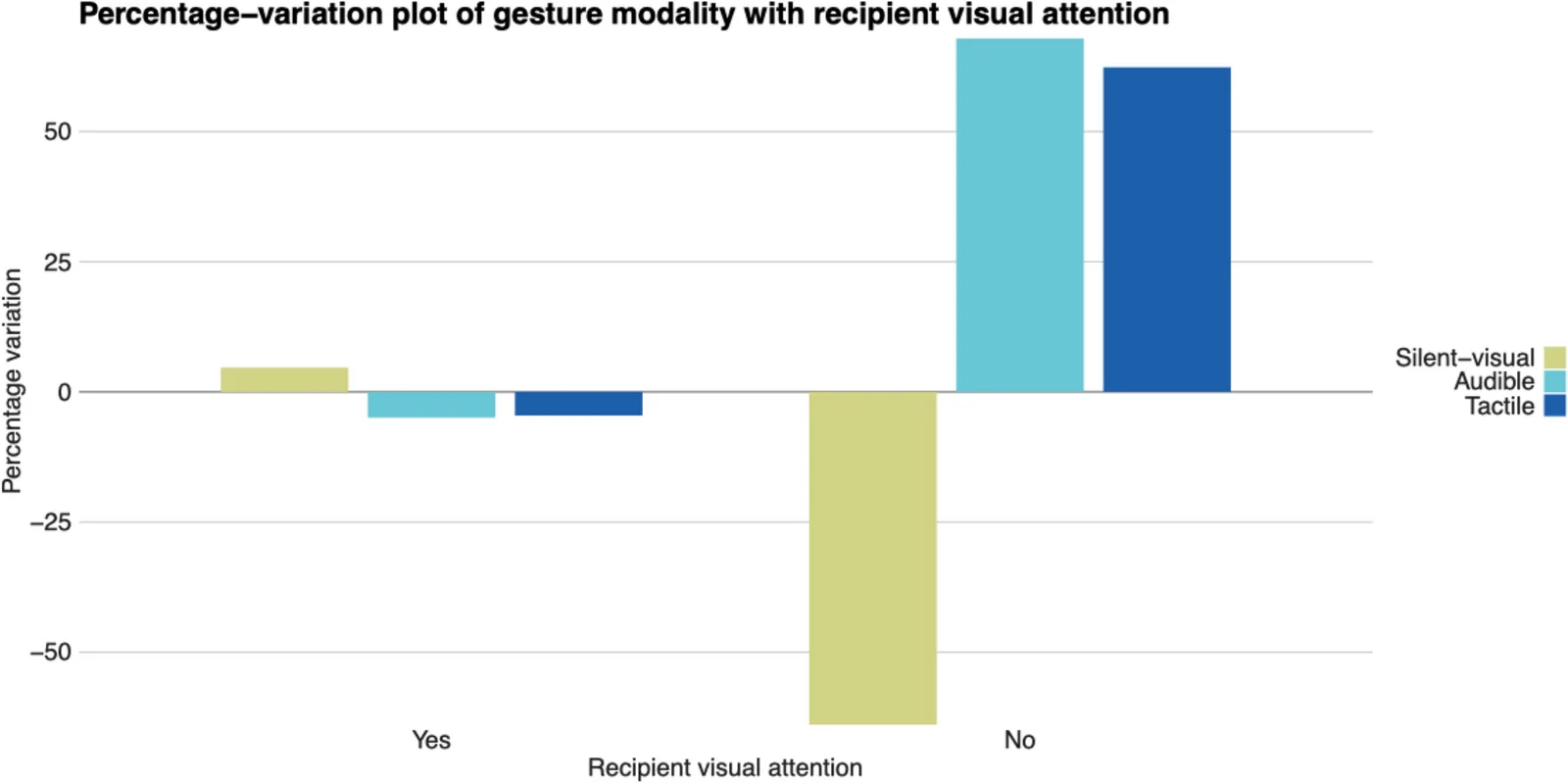
Percentage–variation plot of gesture modality with recipient visual attention in the identified dataset. The plot illustrates how signallers modulate their use of gesture sensory modalities depending on whether recipients were visually attentive. Deviations above and below zero indicate a respective increase or decrease in use of a modality relative to its overall distribution in the dataset. ‘Yes’ = recipient visually attentive; ‘No’ = recipient not visually attentive. N=486 gesture tokens (n silent-visual=245, n audible=114, n tactile=127). For the plot of the whole dataset see Figure S3

*Audience checking*: Signallers gestured while visually attending to recipients 97% (522/539) of the time *before* (Binomial test all data: percent expected=0.50, p<0.001; GLMM identified data: estimate=7.469, s.e.=2.283, z=3.271, p=0.001) and 98% (535/546) of the time *during* the bout part (Binomial test all data: percent expected=0.50, P<0.001; GLMM identified data: estimate=7.405, s.e.=2.356, z=3.143, p=0.002; FigureS1).

**Fig. 2:**
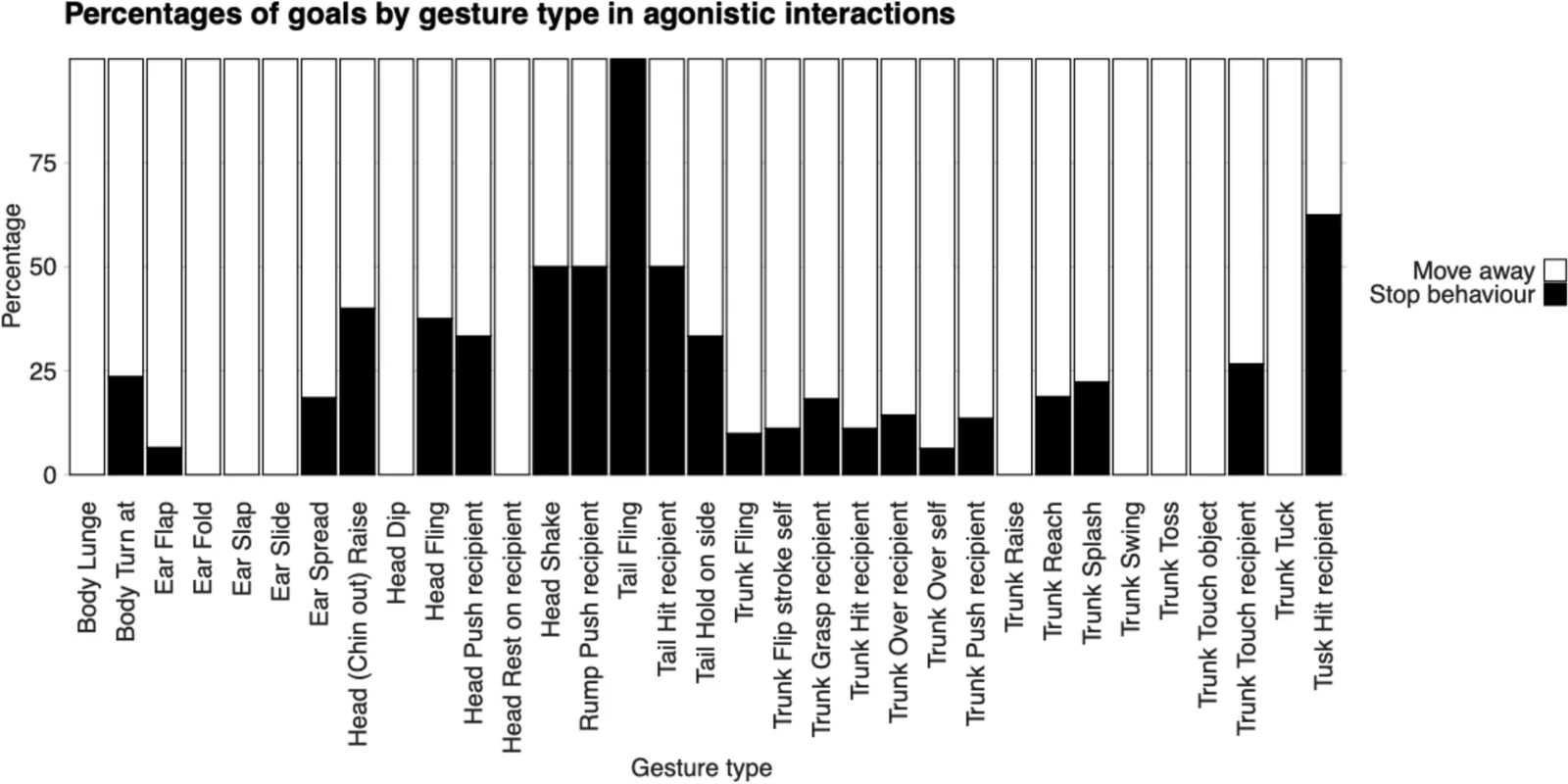
Percentages of (main) goals per gesture type in agonistic interactions. The figure shows the proportion of use of the gesture types for different goals during successful gestural communication events (n tokens=507)

*Sensitivity to recipient attention*: Signallers produced 94% (762/810) of gesture tokens when recipients were visually attentive (Binomial test full dataset: percent expected=0.50, p<0.001; GLMM identified dataset: estimate=6.419, s.e.=1.961, z=3.274, p=0.001; FigureS2). In total, signallers used 97.5% (434/445) of silent-visual, 92% (n=163/178) of audible, and 88% (n=162/184) of tactile gesture tokens when the recipients were visually attentive. At the full dataset level, signallers varied their gesture sensory modality according to the recipient visual attentional state (Chi-squared test: χ^2^=23.442, d.f.=2, p<0.001). However, no significant effect was detected at the identified dataset level, suggesting a potential loss of power due to the reduced sample size or a possible effect of signaller identity (mblogit full-null model comparison: χ^2^=4.871, d.f.=2, p=0.088).

The percentage-variation plots indicated that, when recipients were not visually attentive, signallers substantially (> 50%) increased their use of tactile and audible gesture actions while substantially decreased (> 50%) their use of silent-visual gesture actions, relative to the general distribution of sensory modalities in the dataset (Fig. 1).

*Response waiting* and *persistence*: Signallers showed response waiting after 90% of bout parts (477/531; Binomial test all data: percent expected=0.50, p<0.001; GLMM identified data: estimate=2.905, s.e.=0.521, z=5.577, p<0.001) and persisted gesturing after 83% (334/404) of bout parts (Binomial test all data: percent expected=0.50, p<0.001; GLMM identified data: estimate=2.101, s.e.=0.326, z=6.445, p<0.001; Figure S4).

## Agonistic intentional gestural repertoire and goals

Across 261 gestural communication events including 844 gesture tokens, we recorded a total of 46 general gesture actions expressed as 68 gesture types in the Addo and Marakele elephant populations (Addo: n gestural communication events=184, n gesture tokens=618, n gesture actions=38, n gesture types=51; Marakele: n gestural communication events=77, n gesture tokens=226, n gesture actions=30, n gesture types=44). Out of all gesture types, seven were excluded (Body Lean backward, Ear Hit object, Front limb Stand on, Front limb Swing, Head Lower, Tail Raise, Tusk Touch recipient) as they never led to a recipient reaction meeting the signaller’s apparent goal. 20 gesture types were further excluded as they occurred fewer than three times in this context (See Table S5, Figure S5for full list). We therefore catalogued a total of 32 general gesture actions expressed as 41 gesture types as part of elephants’ intentional gestural repertoire within agonistic interactions (Addo: n gesture types=36, n gesture actions=28; Marakele: n gesture types=30, n gesture actions=24; Table 2). Elephants mostly gestured intentionally with their trunk (47%, n=380/810 gesture tokens) and ears (37%, n=303/810), while other body parts each accounted for <10% of gesture tokens (Table S6). The trunk was the most versatile body part, producing 20 different gesture actions, while the ears were used for six, the head for seven, and the tail for three. Other body parts were used for fewer than three gesture actions (Table 2). Most gesture actions (n=22) were silent-visual, eight were audible, and 11 tactile (Table 2).

**Table 2:** Gestures used intentionally by elephants during agonistic interactions

Gesture action	Gesture type	Definition	Sensory modality	Gesture type frequency	Population	Elephantvoices
Dip	Head Dip	Slightly lowering and raising back up the head	Silent-visual	7	Addo	Head-Dip (not aggressive)
Flap	Ear Flap	Moving the ear(s) back and forth. Repetition of flap movement observed in 4 gesture tokens	Audible	127	Addo, Marakele	Rapid-Ear-Flapping (repeated movement, not aggressive)
Fling	Head Fling	An abrupt movement away from own body and in the direction of the recipient (fling) performed with the head. The energy of the movement is only on the way out	Silent-visual	10	Addo, Marakele	Head-Swipe
	Tail Fling	Flinging the tail in the direction of the recipient. The energy of the movement is only on the way out	Silent-visual	6	Addo, Marakele	
	Trunk Fling	Flinging the trunk in the direction of the recipient. The energy of the movement is only on the way out	Silent-visual	84	Addo, Marakele	Forward-Trunk-Swing
Flip	Trunk Flip	Throwing the trunk upwards causing it to flip over itself (flip)	Silent-visual	5	Addo	
Flip stroke self	Trunk Flip stroke self	Flipping the trunk ending with the trunk stroking itself	Silent-visual	12	Addo	
Fold	Ear Fold	Bending the bottom portion of the ear(s) inward, resulting in a ridge forming across the middle	Silent-visual	18	Addo, Marakele	Ear-Folding
Grasp recipient	Trunk Grasp recipient	Closing the distal portion of the trunk over a recipient’s body part	Tactile	11	Addo	Grasp + body part (e.g., Grasp-Breast, Grasp-Leg, not aggressive)
Hit object	Trunk Hit object	A short hard contact (hit) with an object (i.e., reeds) with the trunk	Audible	3	Addo, Marakele	Bush-Bashing
Hit recipient	Back limb Hit recipient	Hitting recipient with the back limb	Tactile	4	Addo	Kick-Back (not aggressive)
	Tail Hit recipient	Hitting recipient with the tail	Tactile	5	Marakele	Tail-Swatting (aggressive)
	Trunk Hit recipient	Hitting recipient with the trunk. Hit movement repetition observed in three gesture tokens	Tactile	13	Addo, Marakele	Slap
	Tusk Hit recipient	Hitting recipient with the tusk(s)	Tactile	11	Addo, Marakele	Tusking
Hold on side	Tail Hold on side	Moving on side of own body (hold on side) the tail. May have olfactory function of presenting olfactory information in genitals	Silent-visual	12	Addo, Marakele	
	Trunk Hold on side	Holding on side of own body the trunk. Performed by three tuskless females and may represent an adaptation of Trunk Over Self (see below in table) performed on the tusk (see Discussion)	Silent-visual	5	Addo	
Lunge	Body Lunge	Thrusting the body forward towards the recipient	Silent-visual	9	Addo, Marakele	Lunge
Over recipient	Trunk Over recipient	Placing the trunk over recipient’s body (includes extending the whole length of the trunk on the recipient’s back)	Tactile	15	Addo, Marakele	Reach-Over (not aggressive)
Over self	Trunk Over self	Placing the trunk over own body part (i.e., tusk, head)	Silent-visual	31	Addo, Marakele	Casual-Stance (not aggressive)
Push object	Trunk Push object	Making contact with an object and exerting continuous force into it (pushing) using the trunk (i.e., reeds)	Audible	3	Marakele	Bush-Bashing
Push recipient	Head Push recipient	Pushing recipient with the head	Tactile	7	Addo, Marakele	Head-Butt; Pushing
	Rump Push recipient	Pushing recipient with the rump	Tactile	6	Marakele	Lean
	Trunk Push recipient	Pushing recipient with the trunk	Tactile	56	Addo, Marakele	Pushing; Trunk-Push-Down
Raise	Head (Chin out) Raise	Moving vertically up (raise) the head while extending the chin wide outwards	Silent-visual	6	Addo, Marakele	Chin Out
	Trunk Raise	Raising the trunk so that the trunk tip is above the head	Silent-visual	7	Addo, Marakele	Reaching-High with trunk

Elephants gestured to communicate two main goals during successful communication events: “Move away” in 80% (134/168) and “Stop behaviour” in 20% (34/168) of events. Of the 41 gesture types, 33 had at least three gesture tokens observed in successful gestural communication events. Of these, 11 (33%) were exclusively used for “Move Away”, one (3%) exclusively for “Stop behaviour” and 21 (64%) were used for both “Move away” and “Stop behaviour”. “Move away” was the primary main goal (occurring >=70%) for all gesture types except Head (Chin Out) Raise, Head Fling, Head Push recipient, Head Shake, Rump Push recipient, Tail Fling, Tail Hit recipient, Tail Hold on side, Tusk Hit recipient (Fig. 2; Table S7).

“Move away” was most often communicated at recipients who were previously approaching the signaller or a monopolisable resource (28%), feeding on the resource (21%), resting close to the signaller or the resource (22%), or drinking (13%). “Stop behaviour” was most communicated at recipients who were previously approaching the signaller or a monopolisable resource (56%) or drinking (44%) (Table 3).

**Table 3:** Frequencies and percentages of recipient interactional contexts prior to gestural communication events for each main goal (“Move away” and “Stop behaviour”) during agonistic interactions. Only contexts contributing at least three successful gestural communications were included (n events=131)

Recipient prior context	Goal (main)	Number of gestural communication events	Percentage
Agonistic	Move away	7	6.2
Drinking	Move away	15	13.3
Drinking	Stop behaviour	8	44.4
Feeding	Move away	3	2.7
Feeding (monopolisable food)	Move away	24	21.2
Mud bathing	Move away	4	3.5
Resting	Move away	25	22.1
Social context with third party	Move away	3	2.7
Travel	Move away	7	6.2
Travel (move)	Move away	25	22.1
Travel (move)	Stop behaviour	11	55.6

Most gesture types were used flexibly across specific goals within each recipient context, except for Rump Push recipient, used to displace the recipient from reeds, and Tail Fling and Trunk Touch object, used to displace the recipient while he/she was approaching the signaller (Table S8). However, these gesture types were used in the specific context for the specific goal for fewer than 10 tokens.

## Population differences in agonistic gesturing

**Fig. 3:**
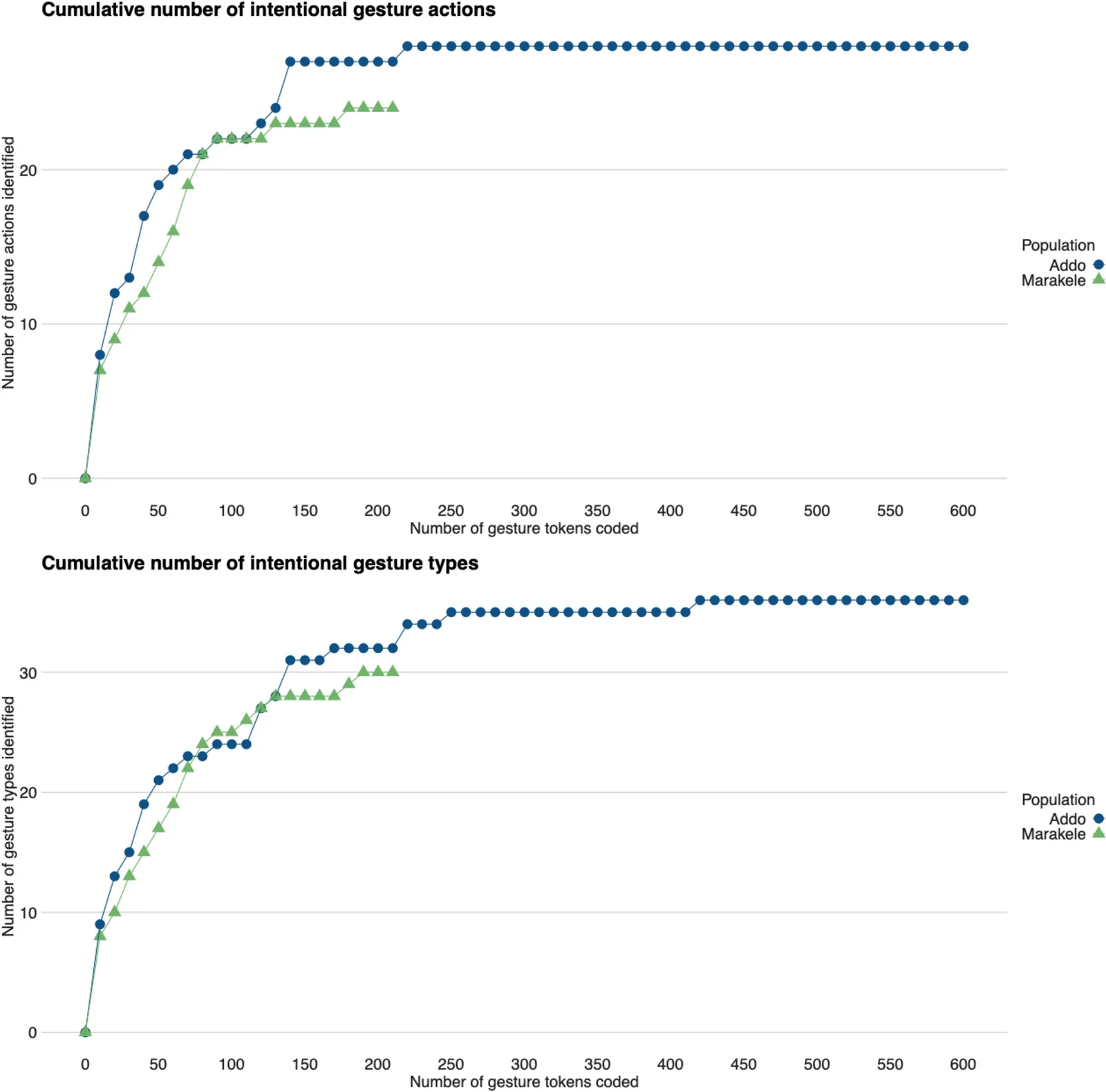
Cumulative number of intentional gesture actions and gesture types used in agonistic interactions by Addo and Marakele elephants. In each population the cumulative number of gesture actions and gesture types (Addo: n gesture actions=28, n gesture types=36, Marakele: n gesture actions=24, n gesture types=30) are respectively plotted against the total number of their gesture tokens coded (n gesture tokens: Addo=600; Marakele=210). See Figure S6 for plot of all identified gesture types

**Fig. 4:**
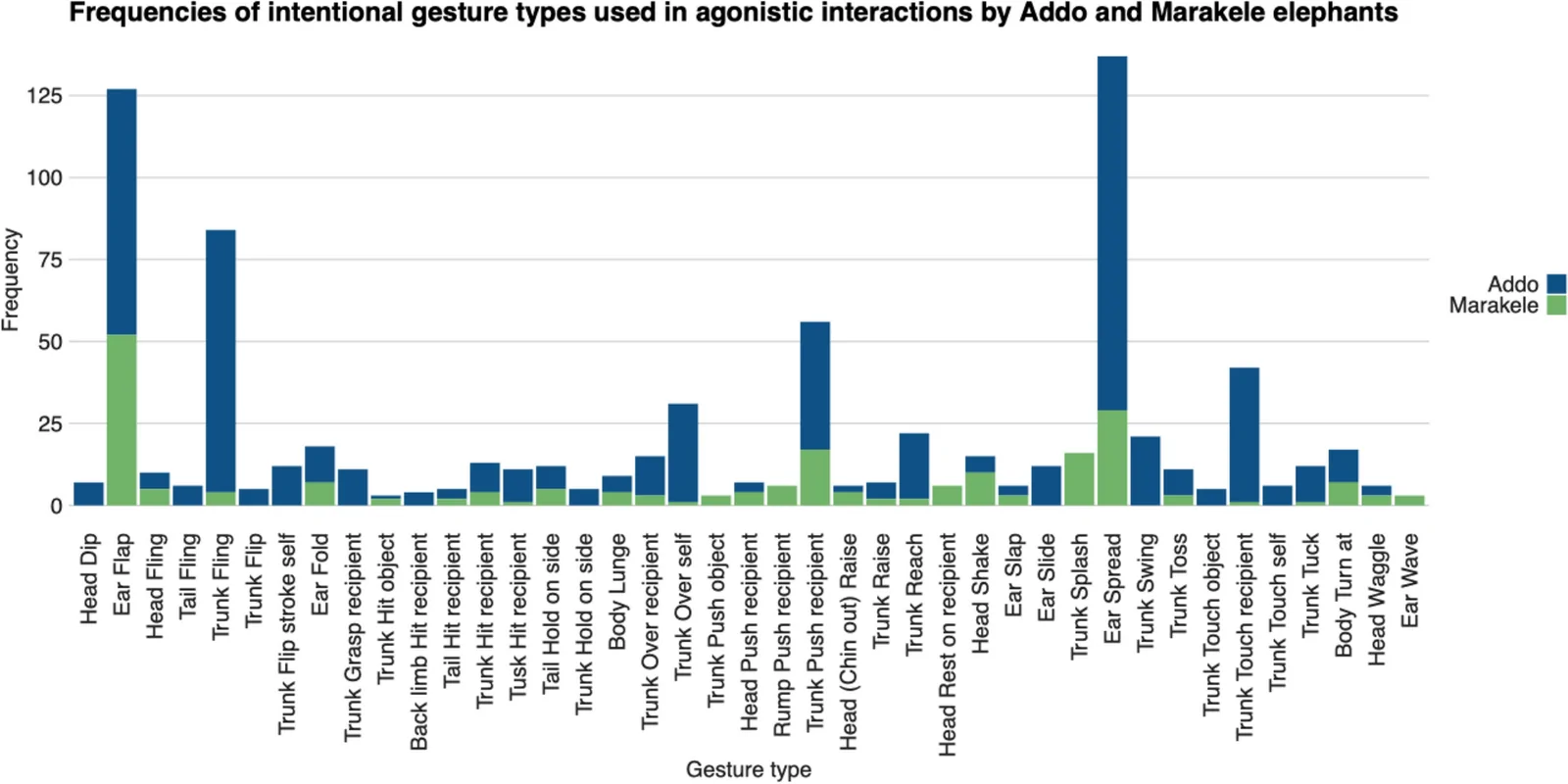
Frequencies of intentional gesture types in agonistic interactions by Addo and Marakele elephants. Gesture types used less than three times were excluded (n gesture tokens=810). For frequencies of all gesture types see Figure S5

The cumulative distribution plots show that the cumulative number of gesture actions as well as gesture types used intentionally during agonistic interactions reach an asymptote in Addo and start approaching an asymptote in Marakele (Fig. 3; see Figure S6 for cumulative frequency plots of all identified gesture actions and gesture types). Addo and Marakele elephants shared 63% (26/41) of their gesture types, while 10 gesture types were only coded in Addo and five only in Marakele (Table 2, Fig. 4). While in Addo sample sizes were similar between males and females (Addo gesture tokens: n males=356, n females=262), in Marakele less than one-third of gesture tokens were from females (Marakele gesture tokens: n males=178, n females=48).

**Fig. 5:**
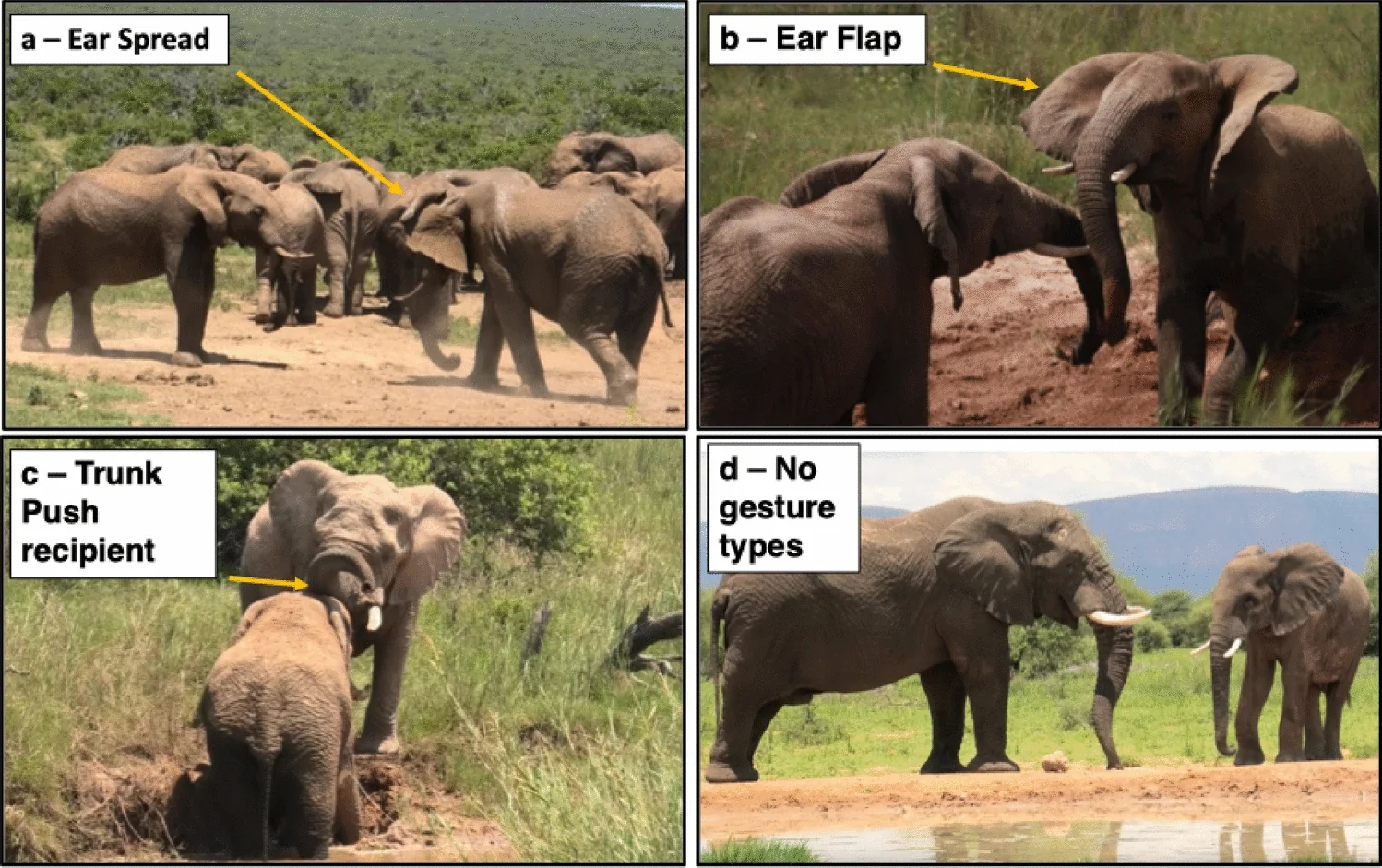
Pictures of the three intentional gesture types most frequently used by both populations in agonistic interactions. Panel a – Ear Spread; Panel b – Ear Flap; Panel c – Trunk Push recipient (See Table 2 for definitions); Panel d – No gesture types: image of two elephants visually attending to each other where no visible gesture types are produced

The “repertoire of regular use” comprised nine gesture types in Addo (Ear Spread, Ear Flap, Trunk Fling, Trunk Over self, Trunk Push recipient, Trunk Touch recipient, Trunk Reach, Trunk Swing, Ear Slide) and nine in Marakele (Body Turn at, Ear Flap, Ear Fold, Ear Spread, Head Rest on recipient, Head Shake, Rump Push recipient, Trunk Push recipient, Trunk Splash) (Table S9). Of these, three were frequently used by both populations (Ear Spread, Ear Flap, Trunk Push recipient) (Fig. 5, Table S9).

In Addo, out of 108 successful gestural communication events, 32% resulted in the recipient moving away from water and 11% stopping their behaviour (e.g., drinking) at the water hole. In Marakele, out of 60 successful communications, 27% resulted in the recipient moving away from the reeds and 7% stopping their behaviour at the reeds (Table 4). Additionally, in Addo, 42% (35/84) of Move away goals consisted in the recipient moving away from the water hole, while 50% (12/24) of Stop behaviour goals consisted in the recipient stopping drinking or approaching the water hole. In Marakele, 32% (16/50) of Move away goals consisted in the recipient moving away from reeds and 4% (2/50) moving away from water, while 40% (4/10) of Stop behaviour goals consisted in the recipient stopping their behaviour at the reeds (Table 4).

**Table 4:** Frequencies and percentages of specific goals of successful gestural communication events of the Addo and Marakele elephant populations in agonistic interactions. Only successful events where the recipient reacted by meeting the signaller’s apparent goal were included (Total n=168). Percentages are calculated within each population

Population	Goal (specific)	Number of gestural communication events	Percentage
Addo	Move away	49	45.4
Addo	Move away from water	35	32.4
Addo	Stop behaviour	12	11.1
Addo	Stop behaviour at water	12	11.1
Marakele	Move away	32	53.3
Marakele	Move away from reeds	16	26.7
Marakele	Move away from water	2	3.3
Marakele	Stop behaviour	6	10
Marakele	Stop behaviour at reeds	4	6.7

The elephant populations did not differ in either the success of their gesturing at achieving goals (Population Outcome GLMM full-null model comparison identified data: χ^2^=1.181, d.f.=1, p=0.277) or their rates of gestural persistence (Population Persistence GLMM full-null model comparison identified data: χ^2^=0.188, d.f.=1, p=0.664).

## Discussion

By exploring elephant agonistic interactions, this study provides the first systematic evidence that wild African savannah elephants gesture intentionally to conspecifics. Elephants visually checked recipients both before and during gesturing and adapted the sensory modality of their gestures based on the recipient’s state of visual attention. After gesturing, they showed response waiting and persisted with further gestures if the recipient did not react in a way that satisfied their goal. Elephants used a large repertoire of gesture types for two main agonistic goals: “Move away” and “Stop behaviour”. Whilst Addo and Marakele elephants largely used the same gesture types (> 60%) to navigate their agonistic interactions, the two populations varied in their repertoires of regular use and in the resources over which they competed. The two populations showed no variation in either the success of their gesture types or the degree of gestural persistence.

Humans use extensive signal repertoires intentionally and flexibly to achieve different goals (Bruner 1981; Dennett 1983; Clark 1996; Tomasello 2010). For example, we may beckon with a hand to ask the recipient to come closer, or to ask for an object. Evidence of goal-directed, intentional communication in non-primate species is growing, but it typically regards the use of a few gesture types associated with a fixed, specific goal (e.g., Pika and Bugnyar 2011; Vail et al. 2013; Ben Mocha and Pika 2019; Ben Mocha et al. 2019). For example, male Arabian babblers use “object presentation” to solicit copulation in females, and coral reef fishes direct their bodies towards prey to solicit cooperative hunting (Vail et al. 2013; Ben Mocha and Pika 2019). In contrast, like humans, some primate species use repertoires of dozens of intentional gesture types (Gupta and Sinha 2019; Molesti et al. 2020; Schel et al. 2022; Villa-Larenas et al. 2024), with apes using up to 90 core gesture actions into 100–150 gesture types across diverse interactional contexts to flexibly communicate up to 15 goals (c.f. Byrne et al. 2017; Mielke et al. 2024). While the size and flexibility of some primates’ gestural repertoires is striking, it has remained unclear whether they are unique in this regard, as robust comparisons with other species remained lacking (see Graham 2024; Aychet et al. 2025).

The GesturalOrigins coding framework was developed to investigate gesture across ape species in a systematic and comparable way (Grund et al. 2023). By adapting this framework to elephants, we investigated their gestural communication in a comparable way to apes, finding that wild African savanna elephants in the Marakele and Addo Elephant National Parks in South Africa intentionally use 32 general gesture actions to produce 41 gesture types with different body parts during agonistic interactions. Four of these gesture types were used only infrequently, but a subset was used often, mirroring findings from primate gestural repertoires (e.g., Pika et al. 2005; Liebal et al. 2006; Schel et al. 2022). While apes and monkeys primarily gesture with their hands or limbs—their most flexible body parts (Hobaiter and Byrne 2011a; Graham et al. 2017; Knox et al. 2019; Mielke et al. 2024; Grund et al. 2025)—elephants used their trunk for almost half of the gesture types. The trunk is a highly dexterous muscular hydrostat used for a variety of purposes, from breathing and vocalizing to feeding and manipulating objects (Sukumar 2003; Fowler and Mikota 2006). Our findings highlight the central role of the trunk in elephant gesturing and invite future studies to explore the mechanics of trunk gesture production. Future research should also investigate how elephants further express their gestural repertoire by integrating other production features such as body part laterality or orientation (c.f., Grund et al. 2023; Mielke et al. 2024).

The two broad goals identified in ape gesturing during agonistic interactions are “Move away” and “Stop behaviour” (Cartmill and Byrne 2010; Hobaiter and Byrne 2014; Graham et al. 2018). Here, elephants gestured to communicate the same goals, with “Move away” occurring in around 80% of successful communications and “Stop behaviour” in 20%. While most gesture types (21/33; 64%) observed during successful communications were used for both goals, the most common goal in the agonistic interactions we studied was for the recipient to “Move away”. These results show evidence of flexible use of elephant gestures for different goals, but also suggest that displacing a recipient from their location or from a resource is particularly important in elephant agonistic interactions (Wittemyer and Getz 2007; O’Connell-Rodwell et al. 2011).

But why do elephants use so many gesture types to communicate two closely related goals? A similar pattern has been reported in eastern chimpanzees, who use around 20 different gesture types for “Stop behaviour” and 14 for “Move away” (Hobaiter and Byrne 2014). A possible explanation for such redundancy is that these broad goals may yield similar outcomes to the signaller depending on the recipient’s prior behaviour. By examining the recipient’s prior behaviour, we found considerable overlap: both goals were frequently directed at recipients approaching the signaller or engaging in resource-related activities, such as approaching and feeding on monopolisable food items or drinking. This pattern suggests that both goals function to interrupt or prevent behaviours directed towards valuable resources.

Consistent with this suggestion, elephants used most gesture types across more specific goals within each recipient prior interactional context. Very few gesture types appeared to be context- or goal-specific in our dataset: Rump Push recipient was exclusively used to displace recipients feeding on reeds, while Tail Fling and Trunk Touch Object were used only when recipients were approaching the signaller. However, these gesture types were observed only in a few cases. Overall, this pattern indicates that gesture types are not rigidly used by elephants for single goals within specific prior recipient contexts. Therefore, although “Move away” and “Stop behaviour” represent graded or closely related goals, elephants may benefit from a set of signals that allow nuanced expression across different situations (Hobaiter and Byrne 2014). Agonistic interactions carry the inherent risk of escalating into physical aggression (Archer 1988; Huntingford 2013; Wilson et al. 2014; Poole and Granli 2021). Employing a variety of gesture types may help signallers ensure that their intention is effectively communicated, clarified, or even reinforced, without requiring escalation into physical aggression.

In addition, the broad goals “Move away” and “Stop behaviour” may include other, more specific, goals. Although for resource-related goals we added more specific goal categories by specifying the type of resource competed for (e.g., “Move away from water”, “Stop behaviour at water”), further fine-grained distinctions and additional data at the gesture-type level are needed to determine the extent of goal flexibility and/or specificity of elephant gestures. For example, different gesture types may be used to communicate other specific goals such as: “Move away *now”*, “Move away *further*” or “Move *there*”.

The use of various gesture types for the same goal may also reflect different communicative strategies depending on the nature of the relationship between the signaller and the recipient. Elephants live in populations of hundreds or thousands of individuals where they have various social relationships, including close family bonds, alliances, and dominance relationships (Wittemyer and Getz 2007; Evans and Harris 2008; Archie et al. 2011; O’Connell-Rodwell et al. 2011). Hitting or pushing the recipient with their trunks could represent more forceful—potentially risky—gesture types, and thus be employed by dominant individuals towards subordinates, or between close kin or bond partners from whom there is less risk of an aggressive reaction. In other more uncertain relationships, elephants may employ more cautious gesture types avoiding physical contact, such as flapping or spreading their ears. To further disambiguate the goals of elephant agonistic gestures, future studies should explore the communicative interactional context or recipient reaction in greater detail, and consider the social relationship between signallers and recipients (Graham et al. 2022; Fröhlich et al. 2025).

Given evidence that ape populations share gestures across repertoires (Hobaiter and Byrne 2011a; Byrne et al. 2017; Mielke et al. 2024), we predicted substantial overlap between elephant populations as well. Addo and Marakele elephants shared over 60% of the gesture types used in agonistic interactions, while ten gesture types were observed only in Addo and five in Marakele. Whereas in Addo the cumulative number of identified gesture types reached an asymptote, in Marakele it only approached an asymptote. This pattern suggests that while we identified most gesture types used during agonistic interactions in Addo, additional observations in Marakele may reveal further gesture types in this context. Consequently, some gesture types observed exclusively in Addo may also occur in Marakele but remain unsampled. The gesture types recorded only in Marakele may similarly reflect sampling differences. For example, Trunk Push object (i.e., reeds) and Trunk Splash were produced during competition over the reeds inside the river, while Ear Wave was produced by two males in musth, from whom data was not sampled in Addo. Despite overlap at the repertoire level, we predicted that each population would preferentially use particular subsets of gesture types, referred to as the “repertoire of regular use” (Badihi 2024). Consistent with this prediction, Addo and Marakele elephants shared only three (~ 30%) gesture types in their repertoires of regular use—Ear Flap, Ear Spread, and Trunk Push recipient—suggesting local variation in the gesture types most frequently used by each population.

We also compared our repertoire to the long-term, large-scale ElephantVoices database, which is based on decades of research and observations from Kenyan and Mozambican elephant populations (Poole and Granli 2021). Only six gesture types (Trunk Flip, Trunk Flip Stroke Self, Trunk Hold on Side, Trunk Swing, Tail Hold on Side, Tail Fling) were not previously described in the database, likely due to our use of systematic, component-based video coding, which enables fine-grained identification of trunk and tail movements. However, in a previous study, we described Trunk Swing and Tail Hold on Side as part of elephant greeting behaviour (Eleuteri et al. 2024), while Trunk Hold on Side was only produced by females in Addo. Given that almost all females lack tusks in this population, this form may be an adaptation of Trunk Over Self (normally performed on the tusk) used by tuskless individuals. Future studies should systematically explore the presence of this gesture type in other tuskless populations to understand the possible origin of this form. Additionally, some of the gesture types we identified were not classified as part of ‘aggressive behaviour’ in the ElephantVoices database; however, our definition of agonistic interactions included displacements and resource competition, in addition to more overtly aggressive interactions represented in the database (Poole and Granli 2021). Many of our gesture types were also reported as agonistic behaviours in a study on male elephant dominance dynamics in Namibia (O’Connell-Rodwell et al. 2011). Given the similarities between our two South African populations, and the overlap in gesture forms with behaviours reported in Kenyan, Mozambican, and Namibian elephants, our findings suggest a largely shared, species-typical repertoire of gestures used in agonistic interactions (Hobaiter and Byrne 2011a; Byrne et al. 2017; Grund et al. 2025), with potential variation in the populations’ repertoires of regular use.

We also found variation in the general frequency of agonistic behaviour in Addo and Marakele elephants. Over 30% of recorded interactions in Addo involved agonistic interactions, especially over water, and around 3% physical aggression, notably higher rates than the ~7% agonistic interactions and 0.1% physical aggression recorded in Marakele. In both populations, data were primarily collected from elephants interacting at water resources (the river in Marakele and artificial waterholes in Addo). Taken together, our results indicate that Addo elephants engage more frequently in agonistic interactions, especially over access to water, and that they regularly use gestures to avoid escalation into physical aggression. However, because we used all-occurrence rather than focal sampling, we could not systematically quantify inter-individual variation in agonistic or gestural behaviour and cannot exclude the possibility that some particularly aggressive individuals contributed disproportionately to the Addo sample, leading to an apparent population difference. Nevertheless, agonistic interactions involving different elephants were routinely observed throughout the day at Addo waterholes, and the substantial difference between the populations suggests that specific individuals are unlikely to fully account for the observed population difference. Future studies integrating individual focal data are needed to determine population-level differences in agonistic behaviour.

Consistent with our findings, Addo has the highest reported rates of fatal aggression between male elephants in Africa (Gough et al. 2008). Previous studies have suggested that their high fatal aggression rate may be due to the disproportionately large number of males relative to the size of the park, leading to high levels of male-male competition over females (Witehouse 2002; Gough et al. 2008). Our findings suggest that competition over water may be another core driver of elephant aggressive behaviour in this population. Further work should quantify the conditions under which competition emerges, identify predictors of severe or fatal physical aggression, and test when and how gesturing reduces escalation in Addo and other populations. Such evidence could inform conservation and management strategies aimed at reducing unsustainable conflict or supporting natural social regulation, particularly in fenced populations.

Our study is a first step, and as such has its limitations. Our sampling periods were relatively brief (Addo: one month; Marakele: three months), as compared to other studies of wild primates (e.g., 8.5 months across groups in Grund et al. 2025; approximately one year in Graham et al. 2017; Gupta and Sinha 2019; Molesti et al. 2020; 1.5 years in Hobaiter and Byrne 2011a). However, those studies examined full species’ repertoires across multiple contexts, which were comprehensively described at around 1500–2000 gesture tokens (Graham et al. 2017; Grund et al. 2025) or 100–150 h of active data collection (Hobaiter and Byrne 2011a; Molesti et al. 2020). Here we coded ~840 gesture tokens within the single context of agonistic interactions in over 90 h of active data collection and comprehensively described the set of intentional gesture types used by Addo elephants. Previous ape studies focusing on a single context have reported comparable sample sizes, despite longer data collection periods (e.g., Fröhlich et al. 2016a, b; Rodrigues et al. 2022). Elephant socio-ecology may explain our comparatively larger sampling in a briefer data collection period: Elephant aggregations are typically larger than those of apes, often comprising tens to hundreds of individuals, and their large size and savannah habitats allow greater visibility than forests, facilitating efficient data collection.

Despite our use of pre-record during video data collection, our approach relied on the observer’s judgement of the start of an interaction, potentially resulting in early subtle gestures being missed and, consequently, in an underestimation of the number of gesture actions and types in the elephants’ repertoire. Alternative methods, such as continuous focal sampling, offer more reliable detection of subtle actions but are challenging to employ in dynamic behavioural contexts. However, as we included gesture types observed at least three times across interactions in the repertoire, we consider it unlikely that a substantial proportion of specific gesture types meeting this inclusion criterion have been entirely missed.

Furthermore, while our study focuses on elephant gestural communication, elephants are known to combine various body movements and vocalizations in different contexts (c.f., Poole and Granli 2021; Eleuteri et al. 2024, 2026). Future research should explore elephants’ intentional use of vocal-gestural combinations, and how communicative flexibility may be enhanced by combining these signalling systems together. Moreover, the extent to which our findings from their agonistic interactions generalise to elephant gestural communication as a whole will require investigation across a broader range of contexts. Lastly, as human observers, we may have overlooked aspects of elephant gesturing or have applied distinctions between gesture forms that that are not meaningful to the elephants themselves. For example, some distinctions in the gesture types or goals we identified may be salient to humans, but not to elephants. Automated classification approaches may represent a promising avenue that may help address these biases and provide a deeper insight into elephant communication across both agonistic and non-agonistic contexts. At this stage, maintaining finer-grained definitions allowed us to explore the full diversity of elephant gestures and identify potential population-level variation in gesture expression and preference of use. In future work across contexts, however, some gesture types may be grouped into broader categories if they do not appear to communicate different goals or to be meaningfully discriminated by elephants themselves (c.f., Hobaiter and Byrne 2017; Mielke et al. 2024).

For a long time, humans—and later some other primates—were considered unique in their ability to communicate intentionally with large and flexible repertoires of signals (Tomasello 2010; Byrne et al. 2017). By extending these abilities to elephants, our study provides evidence for the convergent evolution of intentional, flexible communication systems in a distantly related species facing strong socio-communicative demands, thereby supporting the hypothesis that social complexity drives communicative complexity (Byrne and Bates 2007; Freeberg et al. 2012; Peckre et al. 2019). By systematically describing the gesture types, communicative goals, and rates of elephant agonistic interactions, this study also broadens our understanding of elephant communication and behaviour. We hope these findings will provide valuable insights for elephant management and encourage conservation strategies that incorporate knowledge of elephant communication and behaviour to help protect this endangered keystone species.

## Supplementary Information

Below is the link to the electronic supplementary material.

Supplementary file1 (PDF 870 kb)

Supplementary file2 (MP4 125144 kb)

Supplementary file3 (MP4 65926 kb)

Supplementary file4 (MP4 143944 kb)

Supplementary file5 (MP4 204047 kb)

## Data Availability

The code used for analyses is available on GitHub (https://github.com/VestaEleuteri/Ele-agon/tree/main). Datasets are available from the corresponding author V.E. upon request.
