# Supplementary material for: Elephant gestural communication during agonistic interactions: repertoire, intentionality, and goals
Source: Anim Cogn. 2026 Sep 25;29(1):67. doi: 10.1007/s10071-026-02108-7 (PMC13619774; doi:10.1007/s10071-026-02108-7)
Supplement: Supplementary file 1 [file 10071_2026_2108_MOESM1_ESM.pdf]

Extended Supplementary Material

Title: "**Elephant gestural communication during agonistic interactions: repertoire, intentionality, and goals**"

Journal: *Animal Cognition*

Authors: Vesta Eleuteri[1], Anna Letrari[1], Lucy Bates[2], *Catherine Hobaiter[3], *Angela Stoeger[1,4,5]

[1] Department of Behavioral & Cognitive Biology, University of Vienna, Vienna, Austria
[2] Department of Psychology, University of Portsmouth, Portsmouth, UK
[3] School of Psychology and Neuroscience, University of St Andrews, St Andrews, UK
[4] Zoo Conservation Science Lab, Vienna Zoo, Vienna, Austria

Corresponding authors: Vesta Eleuteri, vesta.eleuteri@gmail.com; Angela Stoeger**,** angela.stoeger-horwath@univie.ac.at

*These authors jointly supervised this study

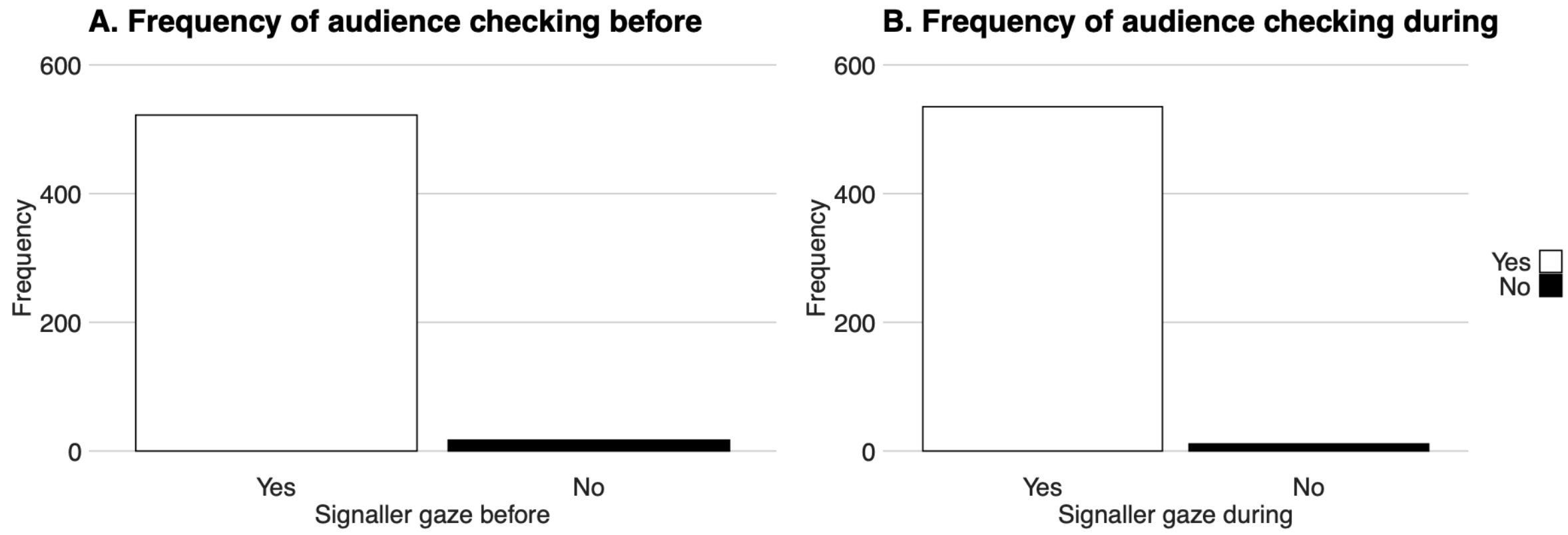

Fig. S1 Frequencies of audience checking at the full dataset level. The plot shows the frequency of bout parts produced while gazing towards the recipient *before* their production (panel A; n=539) and *during* their production (panel B; n=546). ‘Yes’ = signaller gazing; ‘No’ = not gazing

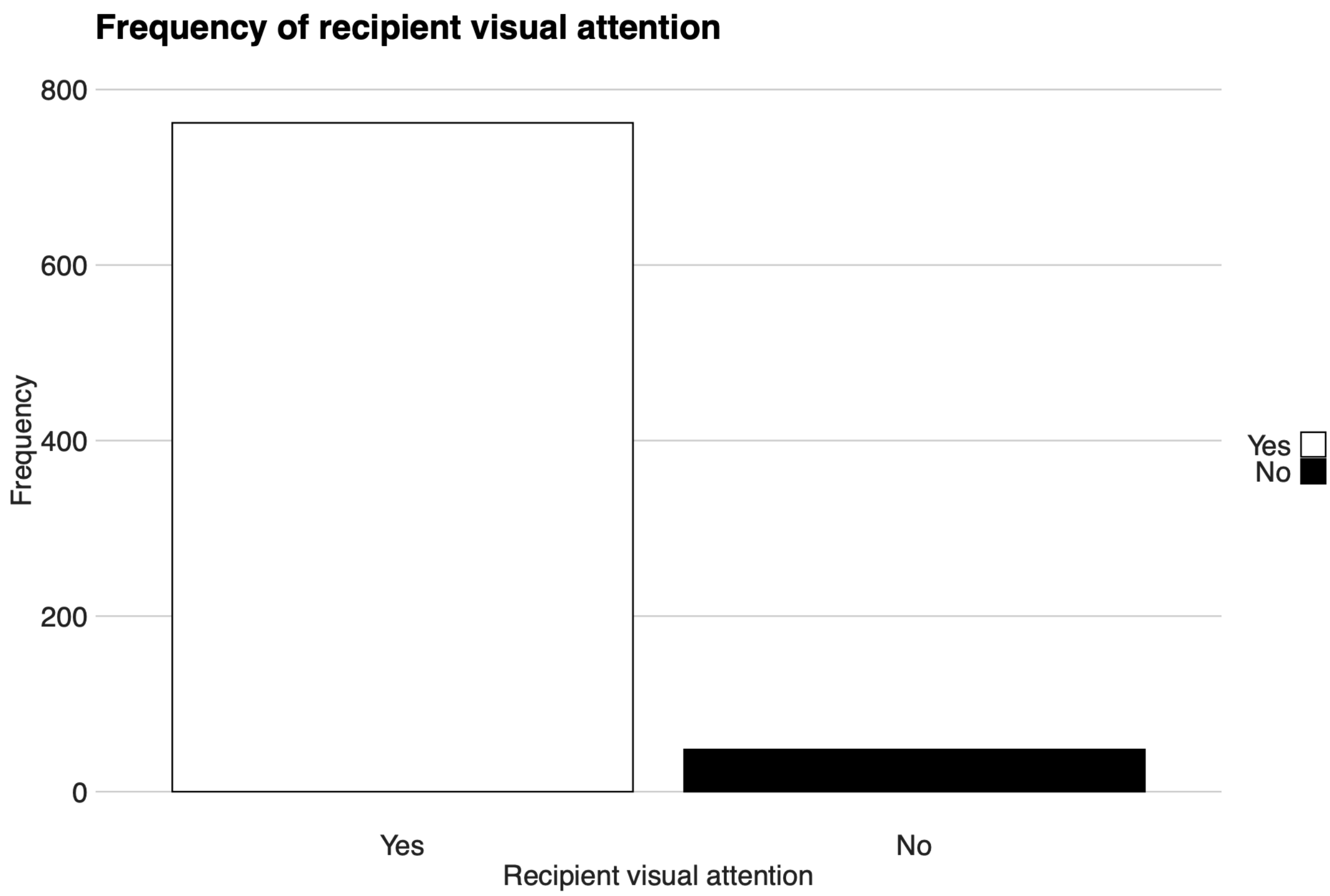

Fig. S2 Frequency of recipient visual attention at the full dataset level. The plot shows the frequency of gesture tokens produced by the signaller while the recipient was visually attentive to them (n=810). ‘Yes’ = recipient visually attentive; ‘No’ = not attentive

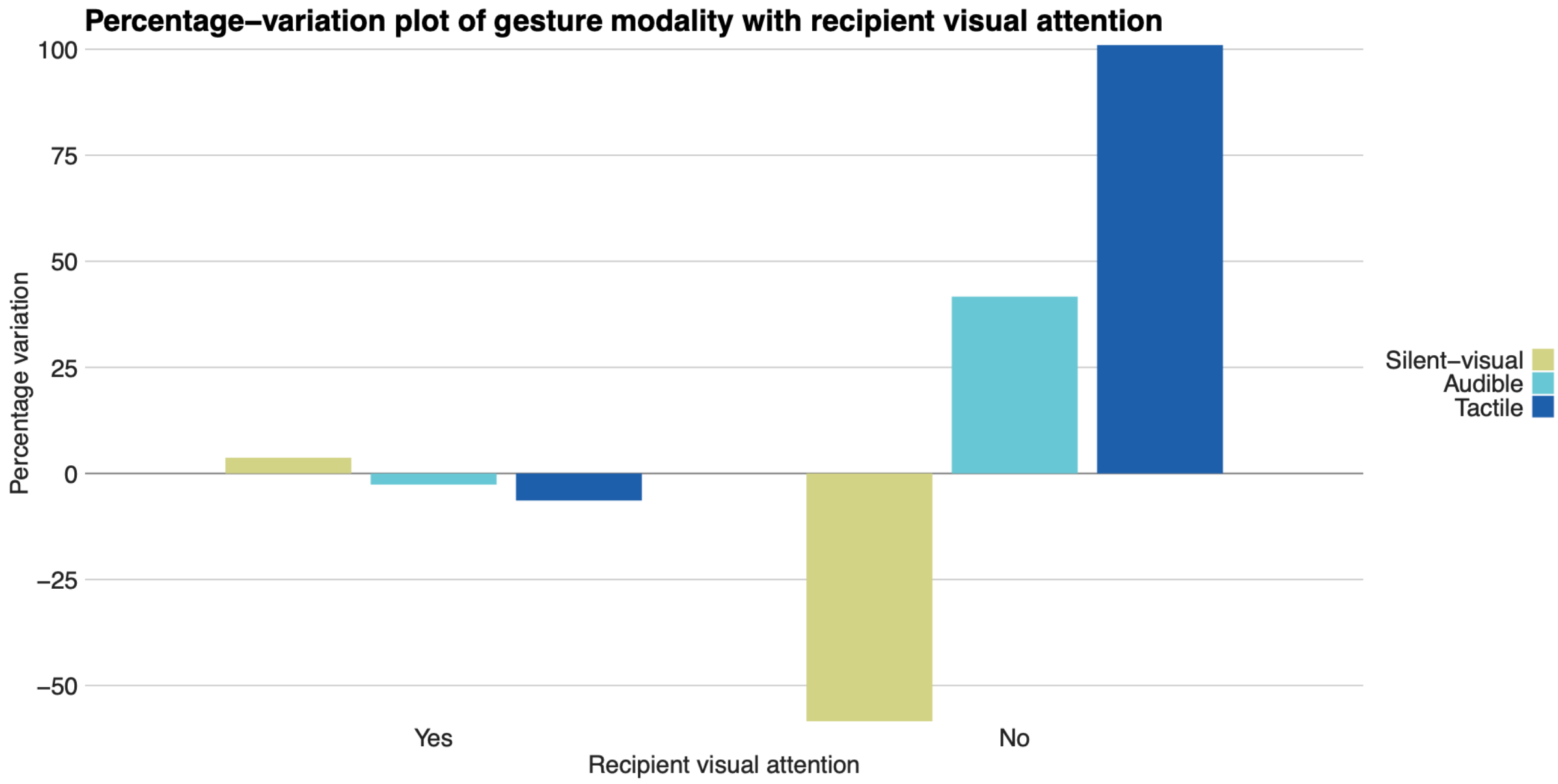

Fig. S3 Percentage–variation plot of gesture modality according to recipient visual attention in the full dataset. The plot illustrates how signallers modulate their use of gesture sensory modalities depending on whether recipients were visually attentive. Deviations above and below zero indicate increase or decrease in use of a modality relative to its overall frequency. "Yes' = recipient visually attentive; 'No' = not attentive. N=807 gesture tokens (n silent-visual=445, n audible=178, n tactile=184)

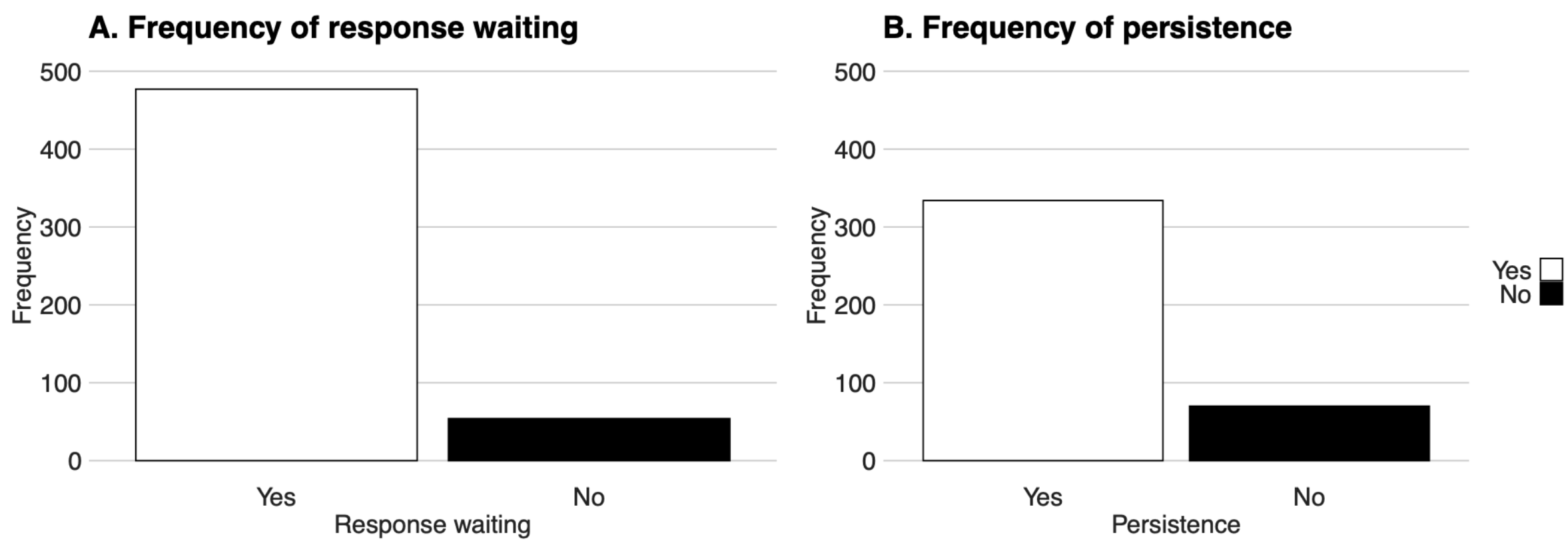

Fig. S4 Frequencies of response waiting and persistence at the whole dataset level. *A. Frequency of response waiting.* The plot shows the frequency of bout parts followed by response waiting by the signaller (n=531). B. *Frequency of persistence*. The plot shows the frequency of bout parts followed by persistence by the signaller when the goal was not met by the recipient (n=404)

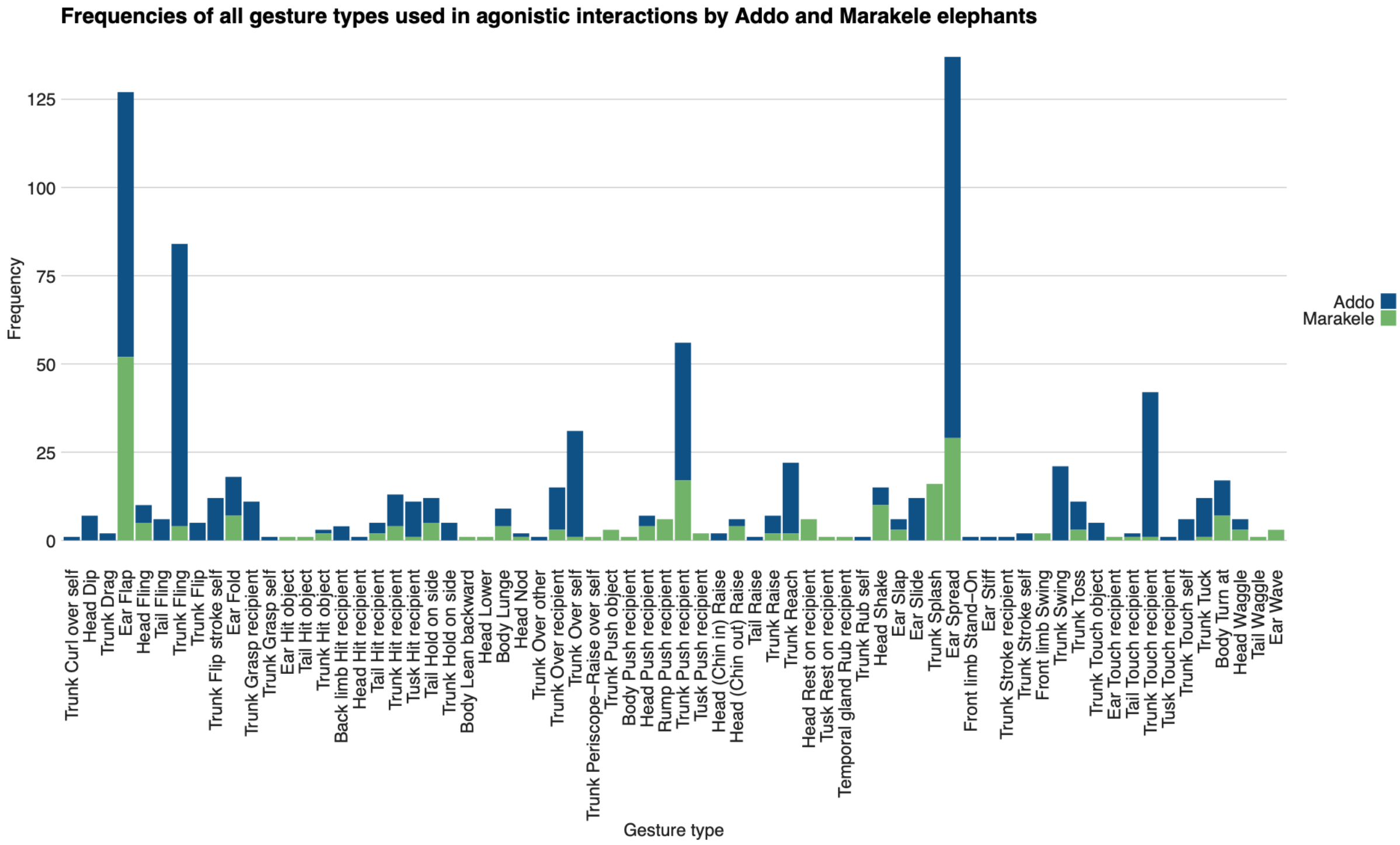

Fig. S5 Frequencies of all gesture types used in agonistic interactions by Addo and Marakele elephants (n tokens=844)

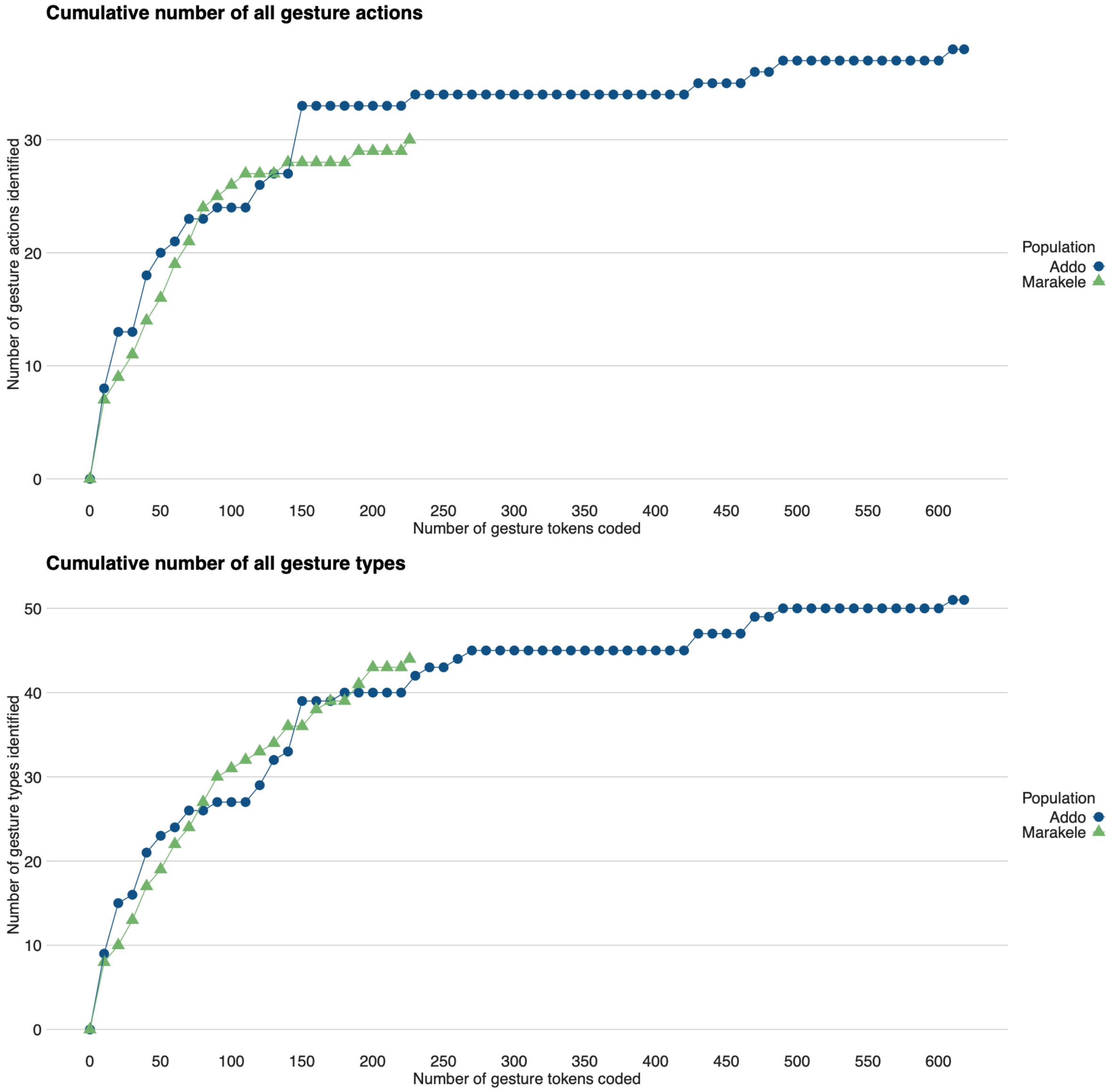

Fig. S6 Cumulative number of all gesture actions and gesture types used in agonistic interactions by Addo and Marakele elephants. In each population the cumulative number of gesture actions and gesture types (Addo: n gesture actions=38, n gesture types=51, Marakele: n gesture actions=30, n gesture types=44) are respectively plotted against the total number of their gesture tokens coded (n tokens: Addo=618; Marakele=226)

Table S1 Variables coded for the study. The main variables "Communication number, Gesture record, and Bout part" appear in bold. Variables under each main variable contain additional information and have the same duration of the main variable or are contained within it (i.e., MAU and Gesture duration)

Variable	Description
**Communication number**	The number of the specific gestural communication event being coded (e.g., VEMC0001). The event started being annotated a few seconds before the first gesture token was produced by the signaller and ended after the recipient reacted by meeting the signaller's goal or, where the goal was not met, when the signaller engaged in a new behaviour or social interaction.
Date	The date when the data was collected.
Video file	The name of the video file coded.
Signaller	The ID of the signaller, where known, or the age category, where unknown (e.g., AM: adult male, AF: adult female, JUVM: juvenile male, JUVF: juvenile female).
Recipient	The ID of the receiver, where known, or the age category, where unknown (e.g., AM: adult male, AF: adult female, JUVM: juvenile male, JUVF: juvenile female).
Signaller prior context	The interactional behavioural context of the signaller before the gestural communication event started (e.g., Traveling, Resting).
Signaller post context	The interactional behavioural context of the signaller after the gestural communication event ended (Traveling, Resting; N.B., Agonistic interactions were marked as "Agonistic" in the signaller post context).
Directed to	Indicates whether there is evidence (typically via head or body orientation) that the gestural communication event was directed to one (e.g., One_dir) or more individuals (e.g., Sev_dir).
Goal	The behavioural reaction of the recipient that results in the signaller stopping gesturing, indicating the recipient's reaction aligned with the signaller's goal (e.g., Move away, Stop behaviour).
Recipient prior context	The interactional behavioural context of the recipient before the gestural communication event started (e.g., Drinking, Travelling).
Recipient post context	The interactional behavioural context of the recipient after the gestural communication event ended (e.g., Resting, Travelling; N.B., if the recipient responded to the signaller with an agonistic interaction, their post context was also marked as "Agonistic").
Communication Comment	Any comment of interest about the gestural communication event.
**Gesture record**	The specific gesture action produced by the signaller (e.g., Reach, Touch) for the gesture token. The duration of the gesture record started being annotated from when the body part started being moved to produce the gesture action until the body part returned to its neutral position.
Gesture record number	The number of the gesture token being coded (e.g., VEMG0001).
MAU and Action duration	The different durations contained within the Gesture action. All gesture actions have a Minimum Action Type (MAU) duration and an Action duration. The MAU represents the smallest possible duration of the gesture action in which it is possible to distinguish the action being produced (e.g., Reach vs Fling). The MAU has the same start of the Gesture record and stops being marked at a given point that allows the action to be discriminated from other gesture actions (See Grund et al., 2023). The Action duration represents the total duration of the gesture action (including any hold or repetition) until the body part starts being moved back to a neutral position. The action duration starts being marked at the end of the MAU and ends when the body part starts being moved to return to a neutral position.

Sequence part	A sequence represents a series of gesture tokens separated by less than one second from the end of the MAU of the previous gesture token and the start of the MAU of the following gesture token. Here we annotated the position of each gesture token within a sequence. E.g., if the sequence consisted of two gesture tokens, we marked the sequence part for the first gesture token as "1_2" (indicating "1st of 2 gesture tokens") and for the second gesture token as "2_2" (indicating "2nd of 2 gesture tokens "). If the gesture token was not part of a sequence (i.e., in the case of single gestures), the sequence part was marked as "1_1".
Body part	The body part used to produce the gesture token (e.g., Ear, Trunk).
Contact part	The signaller's body part a gesture action involving self-contact (e.g., Over self) makes contact with (e.g., Tusk).
Object	Any object used to produce a gesture action involving object use (e.g., Ground, Reeds for Hitting-Object).
Repetition	Marks the number of repetitions for gesture actions consisting in the repetition of a movement (e.g., Swing, which may consist in repeated back and forth swinging of a body part).
Recipient body part	The recipient's body part a gesture action makes contact with or is directed to (e.g., Mouth, Genitals for Touch Recipient).
Recipient visual attention	The recipient's state of visual attention to the gesture token at the onset of its production by the signaller. Elephants have a potential visual field of 313 degrees because their eyes are positioned on the sides of their head (Fowler & Mikota, 2008). Following Eleuteri et al., (2024), we considered visual attention to happen (i.e., Yes) when the signaller's gesture token was in the line of view of the recipient, namely: the recipient was front facing the signaller's gesture token, the recipient's head was at a 90° angle with respect to the token, or the recipient was looking back at the token and the recipient's ears were not obstructing their view. We considered visual attention to not happen (i.e., No) when the signaller's gesture token was not in the line of view of the recipient as described above, and as unknown (i.e., Unk) when the recipient's visual attention could not be assessed (e.g., the recipient was blocked by an object/another elephant).
Duration analysis	Indicates how the gesture token durations should be treated in the analysis (e.g., Exclude_start, Exclude_end etc).
Gesture Comment	Any comment of interest about the gesture token.
Bout part	A bout represents a series of gesture tokens (or sequences) separated by more than one second from the end of the MAU of the previous gesture token and the start of the MAU of the following gesture token. Here we sequentially coded the parts—whether gesture tokens or sequences—making up a bout (i.e., bout parts). E.g., if the bout included two parts, we marked the first bout part as "1_2" (indicating "1st of 2 bout parts"), while the second as "2_2 (indicating "2nd of 2 bout parts"). If there was only one bout in the gestural communication event, we marked the bout part as 1_1 (indicating "1 of 1 bout part"). The duration of each bout part was annotated from the start of the first gesture token to the end of the last gesture token comprised within the bout part. For example, for a bout part comprising a sequence of two gesture tokens, there would be only one bout part annotation spanning both tokens. Bout part was marked as "Unclear" where the presence of previous bout parts could not be assessed (e.g., where a gestural communication event potentially started before the recording began or where signaller visibility was obstructed).
Gaze before	The signaller's state of visual attention towards the recipient at the *onset* of the production of the bout part. We considered signaller visual attention to happen

	(i.e., Yes) when the recipient was in the line of view of the signaller, namely: the signaller was front facing the recipient, the signaller's head was at a 90° angle with respect to the recipient, or the signaller was looking back at the recipient and the ears were not obstructing their view. Signaller visual attention was considered not happen (i.e., No) when the recipient was not in the line of view of the signaller as described above, or as unknown (i.e., Unk) when the signaller's visual attention could not be assessed (e.g., the signaller's head was blocked by an object/another elephant).
Gaze during	The signaller's state of visual attention towards the recipient *during* the production of the bout part. Coded in the same way as "Gaze before".
Response waiting	Indicates whether the signaller paused for at least one second after the production of the bout part (i.e., Yes, No, Unk). The time was measured from the end of the MAU of the last gesture token in the bout part and: a) in case of persistence, the start of the following bout part; b) when the recipient reacted by meeting the signaller's goal; c) if the goal was not met, when the signaller changed their behaviour indicating they ended their communicative attempt.
Persistence	Indicates whether after response waiting the signaller persisted by producing additional bout parts (i.e., Yes, No, Unk).
Goal met	Indicates whether the recipient reacted by satisfying the signaller's goal, as suggested by the signaller ceasing gesturing after the bout part (i.e., "Yes" was coded when the recipient met the signaller's goal. "Partial" where the recipient reacted in a way congruent with the signaller's goal but did not fully complete the reaction, so the signaller kept gesturing. "No" was coded when the recipient did not react by meeting the signaller's goal).
Bout Comment	Any comment of interest about the bout part.

Table S2 Results of inter-observer reliability procedure

Measure	Population	Statistic	Value	Test statistics	p-value
Number of gesture tokens (ICC)	Addo	ICC	0.958	F(12,12)=46.8	<0.001
	Marakele	ICC	0.800	F(5,5)=9.00	0.015
Gesture record	Addo	κ	0.905	z=19	0.000
	Marakele	κ	0.718	z=8.61	0.000
Body part	Addo	κ	0.926	z=10.00	0.000
	Marakele	κ	0.832	z=7.60	<0.001
Recipient visual attention	Addo	κ	0.763	z=7.76	<0.001
	Marakele	κ	0.864	z=5.51	<0.001
Bout part	Addo	κ	0.803	z=14.70	0.000
	Marakele	κ	0.821	z=6.42	<0.001
Gaze before	Addo	κ	0.775	z=7.49	<0.001
	Marakele	κ	0.883	z=5.02	<0.001
Gaze during	Addo	κ	—	Perfect agreement (κ undefined)	—
	Marakele	κ	1.000	z=5.16	<0.001
Response waiting	Addo	κ	0.911	z=6.27	<0.001
	Marakele	κ	1.000	z=4.25	<0.001
Persistence	Addo	κ	0.870	z=5.97	<0.001
	Marakele	κ	0.758	z=3.72	<0.001
Goal met	Addo	κ	0.963	z=8.45	0.000
	Marakele	κ	0.860	z=4.67	<0.001
Goal	Addo	κ	1.000	z=7.92	<0.001
	Marakele	κ	0.910	z=6.29	<0.001

Table S3 Sample sizes. Original sample sizes, trimmed sample sizes after unclear cases were excluded, and individual sample sizes after data from unidentified elephants and identified individuals contributing less than four observations per variable were removed. Our sample sizes are comparable to those reported in Schel et al., 2022

	**Original sample size**	**Trimmed sample size**	**Individual sample size**
Recipient visual attention	847 tokens	810 tokens	489 tokens
Sensitivity to recipient visual attention	847 tokens	807 tokens	486 tokens
Gaze before	562 bout parts	539 bout parts	324 bout parts
Gaze during	562 bout parts	546 bout parts	322 bout parts
Response waiting	562 bout parts	531 bout parts	318 bout parts
Persistence	562 bout parts	404 bout parts	242 bout parts

Table S4 Model stability for all GLMM models. The table shows the model stability estimates and minimum and maximum values of the model stability estimates after removing levels of random effects one at a time

**GLMM model**	**estimate**	**min**	**max**
Gaze before	7.469	5.610	8.691
Gaze during	7.405	5.352	8.611
Recipient visual attention	6.419	5.967	7.587
Response waiting	2.905	2.703	3.068
Persistence	2.101	1.965	2.205
Population Persistence	2.178	2.004	2.327
Population Outcome	1.553	2.015	2.849

Table S5 All identified gesture actions and types used by elephants in agonistic interactions. This table presents the 68 gesture types (within 46 gesture actions) performed by elephants during agonistic interactions. We report their frequencies, definitions, sensory modalities, and the populations in which they were recorded (Addo and/or Marakele), with the number of identified signallers producing each gesture type within each population reported in parentheses. Gesture type frequency includes tokens produced by both identified and unidentified signallers. Gesture types (i.e., Body Lean backward, Ear Hit object, Front limb Stand on, Front limb Swing, Head Lower, Tail Raise, Tusk Touch recipient) that showed no evidence of intentional use by never resulting in a recipient reaction meeting the signaller's apparent goal are highlighted with ~~strikethrough~~. N tokens = 844

**Gesture action**	**Gesture type**	**Definition**	**Sensory modality**	**Gesture type frequency**	**Population**
Curl over self	Trunk Curl over self	Coiling and uncoiling the trunk over a tusk	Silent-visual	1	Addo (1)
Dip	Head Dip	Slightly lowering and raising back up the head.	Silent-visual	7	Addo (2)
Drag	Trunk Drag	Letting the distal portion of the trunk trail along the ground when walking thereby leaving a stretched mark.	Silent-visual	2	Addo (1)
Flap	Ear Flap	Moving the ear(s) back and forth. Repetition of flap movement observed in 4 tokens.	Audible	127	Addo (19), Marakele (11)
Fling	Head Fling	An abrupt movement away from own body and in the direction of the recipient (fling) performed with the head. The energy of the movement is only on the way out.	Silent-visual	10	Addo (4), Marakele (3)
	Tail Fling	Flinging the tail in the direction of the recipient. The energy of the movement is only on the way out.	Silent-visual	6	Addo (1)

	Trunk Fling	Flinging the trunk in the direction of the recipient. The energy of the movement is only on the way out.	Silent-visual	84	Addo (20), Marakele (3)
Flip	Trunk Flip	Throwing the trunk upwards causing it to flip over itself (flip).	Silent-visual	5	Addo (2)
Flip stroke self	Trunk Flip stroke self	Flipping the trunk ending with the trunk stroking itself.	Silent-visual	12	Addo (6)
Fold	Ear Fold	Bending the bottom portion of the ear(s) inward, resulting in a ridge forming across the middle.	Silent-visual	18	Addo (3), Marakele (2)
Grasp recipient	Trunk Grasp recipient	Closing the distal portion of the trunk over a recipient's body part.	Tactile	11	Addo (3)
Grasp self	Trunk Grasp self	Grasping own body part (i.e., tusk) with the trunk.	Silent-visual	1	Addo (1)
Hit object	~~Ear Hit object~~	A short hard contact (hit) with an object (i.e., reeds) with the ear.	Audible	1	Marakele (1)
	Tail Hit object	Hitting an object (i.e., reeds) with the tail.	Audible	1	Marakele (1)
	Trunk Hit object	Hitting an object (i.e., reeds) with the trunk.	Silent-visual	3	Addo (1), Marakele (1)
Hit recipient	Back limb Hit recipient	Hitting recipient with the back limb.	Tactile	4	Addo (1)
	Head Hit recipient	Repeatedly hitting recipient with the head.	Tactile	1	Addo (1)
	Tail Hit recipient	Hitting recipient with the tail.	Tactile	5	Addo (1), Marakele (0)
	Trunk Hit recipient	Hitting recipient with the trunk. Hit movement repetition observed in three tokens.	Tactile	13	Addo (5), Marakele (2)

	Tusk Hit recipient	Hitting recipient with the tusk(s).	Tactile	11	Addo (5), Marakele (0)
Hold on side	Tail Hold on side	Moving on side of own body (hold on side) the tail. May have olfactory function of presenting olfactory information in genitals.	Silent-visual	12	Addo (5), Marakele (2)
	Trunk Hold on side	Holding on side of own body the trunk. Performed by three tuskless females and may represent an adaptation of Trunk Over Self (see below in table) performed on the tusk (see Discussion).	Silent-visual	5	Addo (1)
~~Lean backward~~	~~Body Lean backward~~	Moving the body backward by shifting weight to the rear.	Silent-visual	*1*	Marakele (1)
~~Lower~~	~~Head Lower~~	Bringing the top of the head down below the level of the shoulders.	Silent-visual	1	Marakele (1)
Lunge	Body Lunge	Thrusting the body forward towards the recipient.	Silent-visual	9	Addo (2), Marakele (3)
Nod	Head Nod	Raising and lowering the head.	Silent-visual	2	Addo (1), Marakele (1)
Over other	Trunk Over other	Placing the trunk over a third-party individual's body (includes extending the whole length of the trunk on their back).	Silent-visual	1	Addo (1)
Over recipient	Trunk Over recipient	Same as Trunk Over other but performed on the recipient.	Tactile	15	Addo (3), Marakele (2)
Over self	Trunk Over self	Placing the trunk over own body part (i.e., tusk, head).	Silent-visual	31	Addo (12), Marakele (1)

Periscope-Raise self	Trunk Periscope-Raise over self	Moving the trunk into an "S" shape and raising it so that the trunk tip is above the head.	Silent-visual	1	Marakele (1)
Push object	Trunk Push object	Making contact with an object and exerting continuous force into it (pushing) using the trunk (i.e., reeds).	Audible	3	Marakele (2)
Push recipient	Body Push recipient	Pushing recipient with the central body.	Tactile	1	Marakele (0)
	Head Push recipient	Pushing recipient with the head.	Tactile	7	Addo (1), Marakele (3)
	Rump Push recipient	Pushing recipient with the rump.	Tactile	6	Marakele (1)
	Trunk Push recipient	Pushing recipient with the trunk.	Tactile	56	Addo (7), Marakele (7)
	Tusk Push recipient	Pushing recipient with the tusk(s).	Tactile	2	Marakele (1)
Raise	Head (Chin in) Raise	Moving vertically up (raise) the head while keeping the chin tucked in.	Silent-visual	2	Addo (1)
	Head (Chin out) Raise	Raising the head while extending the chin wide outwards.	Silent-visual	6	Addo (2), Marakele (4)
	~~Tail Raise~~	Raising the tail to more than 90° from the vertical resting position.	Silent-visual	1	Addo (0)
	Trunk Raise	Raising the trunk so that the trunk tip is above the head.	Silent-visual	7	Addo (3), Marakele (1)
Reach	Trunk Reach	Extending the trunk towards the recipient or location.	Silent-visual	22	Addo (3), Marakele (2)

Rest on recipient	Head Rest on recipient	Keeping still on the recipient (rest) the head.	Tactile	6	Marakele (4)
	Tusk Rest on recipient	Resting tusk(s) on the recipient.	Tactile	1	Marakele (1)
Rub recipient	Temporal gland Rub recipient	Repeatedly pressing up and down (rubbing) on recipient's body part own temporal gland.	Tactile	1	Marakele (1)
Rub self	Trunk Rub self	Rubbing own body part (i.e., temporal gland) with trunk.	Silent-visual	1	Addo (1)
Shake	Head Shake	An abrupt lateral movement (shake) of the head causing the ears to slap the neck. Shaking movement repetition observed in one token.	Audible	15	Addo (2), Marakele (8)
Slap	Ear Slap	Hitting ear(s) against own neck.	Audible	6	Addo (1), Marakele (2)
Slide	Ear Slide	Moving up and down the ear(s) while holding them back against the shoulders causing a rasping sound.	Audible	12	Addo (6)
Splash	Trunk Splash	Splashing the trunk, often in the direction of the recipient. Splashing movement repetition observed in 2 tokens.	Audible	16	Marakele (4)
Spread	Ear Spread	Opening the ear(s) full forward.	Silent-visual	137	Addo (35), Marakele (12)
~~Stand on~~	~~Front limb Stand on~~	Standing on an object with the front limb.	Silent-visual	1	Addo (1)
Stiff	Ear Stiff	Slightly opening the ears up to 45°.	Silent-visual	1	Addo (1)

Stroke recipient	Trunk Stroke recipient	Moving the trunk gently along (stroking) a recipient's body part.	Tactile	1	Addo (1)
Stroke self	Trunk Stroke self	Stroking own body part (i.e., trunk) with the trunk.	Silent-visual	2	Addo (1)
Swing	~~Front limb Swing~~	Smooth continuous movement back and forth (swinging) of the front limb. The energy of the movement is both on the way out and on the way back in.	Silent-visual	2	Marakele (2)
	Trunk Swing	Swinging the trunk back and forth. Differs from Trunk Fling in that the energy of movement is both on way out and back in. Swinging movement repetition observed in 14 tokens.	Silent-visual	21	Addo (5)
Toss	Trunk Toss	An abrupt movement in the air of the trunk without controlling its direction. Differs from Trunk Fling in that it is not directed at the recipient. Tossing movement repetition observed in three tokens.	Silent-visual	11	Addo (3), Marakele (3)
Touch object	Trunk Touch object	Making contact (touch) with an object (i.e., ground) using the trunk.	Silent-visual	5	Addo (1)
Touch recipient	Ear Touch recipient	Touching recipient with the ear.	Tactile	1	Marakele (0)
	Tail Touch recipient	Touching recipient with the tail.	Tactile	2	Addo (1), Marakele (0)

	Trunk Touch recipient	Touching recipient with the trunk.	Tactile	42	Addo (9), Marakele (1)
	~~Tusk Touch recipient~~	Touching recipient with the tusk.	Tactile	1	Addo (1)
Touch self	Trunk Touch self	Conspicuously touching own body (i.e., trunk, tusk) with the trunk. Cases that may represent self-soothing actions were not coded.	Silent-visual	6	Addo (2)
Tuck	Trunk Tuck	Inserting the distal portion of the trunk in-between the base of the trunk and the tusk.	Silent-visual	12	Addo (5), Marakele (1)
Turn at	Body Turn at	Abruptly orienting the front of the body to face the recipient.	Silent-visual	17	Addo (8), Marakele (2)
Waggle	Head Waggle	Alternating up-and-down motion on each side (waggling) performed with the head. Waggling movement repetition observed in one token.	Silent-visual	6	Addo (3), Marakele (3)
	Tail Waggle	Repeatedly waggling the tail raising it up and down from side to side along the rump.	Silent-visual	1	Marakele (1)
Wave	Ear Wave	Moving the upper part of the ear(s) forward and backward, generating a wave-like motion along the ear. Performed by two musth males. May have olfactory function of wafting the temporal gland smell toward the recipient.	Audible	3	Marakele (2)

Table S6 Frequency of use of body parts to produce the 41 intentional gesture types (n tokens=810)

Body part	Frequency	Percentage
Back limb	4	0.49
Body	26	3.21
Ear	303	37.41
Head	51	6.30
Head (Chin out)	6	0.74
Rump	6	0.74
Tail	23	2.84
Trunk	380	46.91
Tusk	11	1.36

Table S7 Frequencies and percentages of agonistic main goals per gesture type (n tokens=507)

Gesture type	Goal	Frequency	Percentage
Body Lunge	Move away	9	100
Body Turn at	Move away	13	76.47
Body Turn at	Stop behaviour	4	23.53
Ear Flap	Move away	72	93.51
Ear Flap	Stop behaviour	5	6.49
Ear Fold	Move away	12	100
Ear Slap	Move away	5	100
Ear Slide	Move away	8	100
Ear Spread	Move away	75	81.52
Ear Spread	Stop behaviour	17	18.48
Head (Chin out) Raise	Move away	3	60
Head (Chin out) Raise	Stop behaviour	2	40
Head Dip	Move away	6	100
Head Fling	Move away	5	62.50
Head Fling	Stop behaviour	3	37.50
Head Push recipient	Move away	4	66.67
Head Push recipient	Stop behaviour	2	33.33
Head Rest on recipient	Move away	5	100
Head Shake	Move away	2	50
Head Shake	Stop behaviour	2	50
Rump Push recipient	Move away	3	50

Rump Push recipient	Stop behaviour	3	50
Tail Fling	Stop behaviour	6	100
Tail Hit recipient	Move away	2	50
Tail Hit recipient	Stop behaviour	2	50
Tail Hold on side	Move away	6	66.67
Tail Hold on side	Stop behaviour	3	33.33
Trunk Fling	Move away	55	90.16
Trunk Fling	Stop behaviour	6	9.84
Trunk Flip stroke self	Move away	8	88.89
Trunk Flip stroke self	Stop behaviour	1	11.11
Trunk Grasp recipient	Move away	9	81.82
Trunk Grasp recipient	Stop behaviour	2	18.18
Trunk Hit recipient	Move away	8	88.89
Trunk Hit recipient	Stop behaviour	1	11.11
Trunk Over recipient	Move away	6	85.71
Trunk Over recipient	Stop behaviour	1	14.29
Trunk Over self	Move away	15	93.75
Trunk Over self	Stop behaviour	1	6.25
Trunk Push recipient	Move away	32	86.49
Trunk Push recipient	Stop behaviour	5	13.51
Trunk Raise	Move away	6	100
Trunk Reach	Move away	13	81.25
Trunk Reach	Stop behaviour	3	18.75
Trunk Splash	Move away	7	77.78
Trunk Splash	Stop behaviour	2	22.22
Trunk Swing	Move away	14	100
Trunk Toss	Move away	3	100
Trunk Touch object	Move away	5	100
Trunk Touch recipient	Move away	11	73.33
Trunk Touch recipient	Stop behaviour	4	26.67
Trunk Tuck	Move away	3	100
Tusk Hit recipient	Move away	3	37.50

Tusk Hit recipient	Stop behaviour	5	62.50

Table S8 Frequencies and percentages of gesture types used for specific goals ("move away from water" and "stop behaviour at water") across recipient interactional contexts prior to gestural communication events in the Addo and Marakele elephant populations during agonistic interactions. Only gesture types contributing at least three gesture tokens were included. Percentages are calculated within each gesture type (n = 417 tokens).

Gesture type	Recipient prior context	Goal (specific)	Frequency	Percentage
Body Lunge	Drinking	Move away from water	1	14.29
Body Lunge	Feeding (monopolisable food)	Move away	1	14.29
Body Lunge	Resting	Move away	2	28.57
Body Lunge	Social context with third party	Move away	1	14.29
Body Lunge	Travel	Move away	1	14.29
Body Lunge	Travel (move)	Move away from water	1	14.29
Body Turn at	Agonistic	Move away from water	1	6.67
Body Turn at	Drinking	Move away from water	1	6.67
Body Turn at	Feeding (monopolisable food)	Move away from reeds	2	13.33
Body Turn at	Resting	Move away	1	6.67
Body Turn at	Resting	Move away from water	1	6.67
Body Turn at	Travel	Move away	3	20
Body Turn at	Travel (move)	Move away	2	13.33
Body Turn at	Travel (move)	Move away from water	2	13.33
Body Turn at	Travel (move)	Stop behaviour	1	6.67
Body Turn at	Travel (move)	Stop behaviour at water	1	6.67
Ear Flap	Agonistic	Move away	10	14.49
Ear Flap	Agonistic	Move away from water	2	2.90
Ear Flap	Drinking	Move away from water	2	2.90
Ear Flap	Feeding	Move away	7	10.14
Ear Flap	Feeding (monopolisable food)	Move away	5	7.25
Ear Flap	Feeding (monopolisable food)	Move away from reeds	14	20.29
Ear Flap	Mud bathing	Move away	7	10.14
Ear Flap	Resting	Move away	4	5.80
Ear Flap	Travel	Move away	2	2.90

Ear Flap	Travel (move)	Move away	10	14.49
Ear Flap	Travel (move)	Move away from water	3	4.35
Ear Flap	Travel (move)	Stop behaviour	2	2.90
Ear Flap	Travel (move)	Stop behaviour at water	1	1.45
Ear Fold	Drinking	Move away from water	1	10
Ear Fold	Feeding (monopolisable food)	Move away from reeds	2	20
Ear Fold	Resting	Move away	1	10
Ear Fold	Travel	Move away	4	40
Ear Fold	Travel (move)	Move away	2	20
Ear Slap	Feeding (monopolisable food)	Move away	1	33.33
Ear Slap	Travel (move)	Move away	2	66.67
Ear Slide	Agonistic	Move away	1	16.67
Ear Slide	Drinking	Move away from water	1	16.67
Ear Slide	Travel (move)	Move away	4	66.67
Ear Spread	Agonistic	Move away	3	4.05
Ear Spread	Drinking	Move away from water	5	6.76
Ear Spread	Drinking	Stop behaviour at water	4	5.41
Ear Spread	Feeding	Move away	1	1.35
Ear Spread	Feeding (monopolisable food)	Move away	3	4.05
Ear Spread	Feeding (monopolisable food)	Move away from reeds	4	5.41
Ear Spread	Mud bathing	Move away	1	1.35
Ear Spread	Resting	Move away	10	13.51
Ear Spread	Resting	Move away from water	5	6.76
Ear Spread	Social context with third party	Move away	1	1.35
Ear Spread	Travel	Move away	8	10.81
Ear Spread	Travel (move)	Move away	15	20.27
Ear Spread	Travel (move)	Move away from water	8	10.81
Ear Spread	Travel (move)	Stop behaviour	2	2.70
Ear Spread	Travel (move)	Stop behaviour at reeds	1	1.35
Ear Spread	Travel (move)	Stop behaviour at water	3	4.05
Head (Chin out) Raise	Travel	Move away	1	33.33

Head (Chin out) Raise	Travel (move)	Stop behaviour	1	33.33
Head (Chin out) Raise	Travel (move)	Stop behaviour at reeds	1	33.33
Head Dip	Agonistic	Move away	2	33.33
Head Dip	Resting	Move away	3	50
Head Dip	Travel (move)	Move away	1	16.67
Head Fling	Agonistic	Move away	1	14.29
Head Fling	Drinking	Stop behaviour at water	2	28.57
Head Fling	Feeding	Move away	1	14.29
Head Fling	Feeding (monopolisable food)	Move away	1	14.29
Head Fling	Resting	Move away from water	1	14.29
Head Fling	Social context with third party	Move away	1	14.29
Head Push recipient	Drinking	Move away from water	1	16.67
Head Push recipient	Drinking	Stop behaviour at water	2	33.33
Head Push recipient	Feeding (monopolisable food)	Move away	1	16.67
Head Push recipient	Feeding (monopolisable food)	Move away from reeds	1	16.67
Head Push recipient	Mud bathing	Move away	1	16.67
Head Rest on recipient	Drinking	Move away from water	1	20
Head Rest on recipient	Feeding (monopolisable food)	Move away	1	20
Head Rest on recipient	Feeding (monopolisable food)	Move away from reeds	1	20
Head Rest on recipient	Mud bathing	Move away	2	40
Rump Push recipient	Feeding (monopolisable food)	Move away from reeds	3	100
Tail Fling	Travel (move)	Stop behaviour	6	100
Tail Hit recipient	Feeding (monopolisable food)	Move away from reeds	2	50
Tail Hit recipient	Travel (move)	Stop behaviour	2	50
Tail Hold on side	Drinking	Move away from water	3	33.33
Tail Hold on side	Feeding (monopolisable food)	Move away from reeds	1	11.11
Tail Hold on side	Resting	Move away	1	11.11
Tail Hold on side	Travel (move)	Move away	1	11.11
Tail Hold on side	Travel (move)	Stop behaviour	2	22.22
Tail Hold on side	Travel (move)	Unk	1	11.11

Trunk Fling	Agonistic	Move away	18	34.62
Trunk Fling	Drinking	Move away from water	4	7.69
Trunk Fling	Drinking	Stop behaviour at water	1	1.92
Trunk Fling	Feeding (monopolisable food)	Move away	1	1.92
Trunk Fling	Feeding (monopolisable food)	Move away from reeds	1	1.92
Trunk Fling	Resting	Move away	13	25
Trunk Fling	Resting	Move away from water	1	1.92
Trunk Fling	Social context with third party	Move away	1	1.92
Trunk Fling	Travel (move)	Move away	11	21.15
Trunk Fling	Travel (move)	Stop behaviour	1	1.92
Trunk Flip stroke self	Agonistic	Move away	1	33.33
Trunk Flip stroke self	Resting	Move away	1	33.33
Trunk Flip stroke self	Travel (move)	Stop behaviour	1	33.33
Trunk Grasp recipient	Drinking	Stop behaviour at water	2	18.18
Trunk Grasp recipient	Resting	Move away from water	8	72.73
Trunk Grasp recipient	Travel (move)	Move away	1	9.09
Trunk Hit recipient	Agonistic	Move away	2	28.57
Trunk Hit recipient	Drinking	Stop behaviour at water	1	14.29
Trunk Hit recipient	Feeding (monopolisable food)	Move away from reeds	2	28.57
Trunk Hit recipient	Travel	Move away from reeds	1	14.29
Trunk Hit recipient	Travel (move)	Move away from water	1	14.29
Trunk Over recipient	Agonistic	Move away	1	16.67
Trunk Over recipient	Drinking	Stop behaviour at water	1	16.67
Trunk Over recipient	Feeding (monopolisable food)	Move away from reeds	2	33.33
Trunk Over recipient	Mud bathing	Move away	1	16.67
Trunk Over recipient	Travel (move)	Move away	1	16.67
Trunk Over self	Drinking	Move away from water	2	14.29

Trunk Over self	Drinking	Stop behaviour at water	1	7.14
Trunk Over self	Resting	Move away	4	28.57
Trunk Over self	Resting	Move away from water	1	7.14
Trunk Over self	Travel (move)	Move away	5	35.71
Trunk Over self	Travel (move)	Move away from water	1	7.14
Trunk Push recipient	Drinking	Move away from water	4	14.29
Trunk Push recipient	Drinking	Stop behaviour at water	2	7.14
Trunk Push recipient	Feeding (monopolisable food)	Move away	2	7.14
Trunk Push recipient	Feeding (monopolisable food)	Move away from reeds	7	25
Trunk Push recipient	Mud bathing	Move away	3	10.71
Trunk Push recipient	Resting	Move away	4	14.29
Trunk Push recipient	Resting	Move away from water	4	14.29
Trunk Push recipient	Travel (move)	Move away	1	3.57
Trunk Push recipient	Travel (move)	Move away from water	1	3.57
Trunk Raise	Agonistic	Move away	1	16.67
Trunk Raise	Feeding (monopolisable food)	Move away from reeds	1	16.67
Trunk Raise	Resting	Move away	2	33.33
Trunk Raise	Travel (move)	Move away	2	33.33
Trunk Reach	Agonistic	Move away	1	8.33
Trunk Reach	Feeding (monopolisable food)	Move away	1	8.33
Trunk Reach	Resting	Move away	4	33.33
Trunk Reach	Travel (move)	Move away	6	50
Trunk Splash	Feeding (monopolisable food)	Move away	2	28.57
Trunk Splash	Feeding (monopolisable food)	Move away from reeds	4	57.14
Trunk Splash	Travel (move)	Stop behaviour	1	14.29
Trunk Swing	Agonistic	Move away	9	64.29
Trunk Swing	Resting	Move away	1	7.14
Trunk Swing	Travel (move)	Move away	3	21.43
Trunk Swing	Travel (move)	Move away from water	1	7.14
Trunk Toss	Resting	Move away	1	33.33

Trunk Toss	Resting	Move away from water	1	33.33
Trunk Toss	Travel (move)	Move away	1	33.33
Trunk Touch object	Travel (move)	Move away	5	100
Trunk Touch recipient	Agonistic	Move away	3	27.27
Trunk Touch recipient	Drinking	Move away from water	2	18.18
Trunk Touch recipient	Drinking	Stop behaviour at water	3	27.27
Trunk Touch recipient	Feeding	Move away	1	9.09
Trunk Touch recipient	Resting	Move away	1	9.09
Trunk Touch recipient	Travel (move)	Move away	1	9.09
Tusk Hit recipient	Drinking	Stop behaviour at water	2	33.33
Tusk Hit recipient	Feeding (monopolisable food)	Move away from reeds	1	16.67
Tusk Hit recipient	Travel	Move away	1	16.67
Tusk Hit recipient	Travel (move)	Move away	1	16.67
Tusk Hit recipient	Travel (move)	Stop behaviour	1	16.67

Table S9 The repertoires of regular use of agonistic gesture types by Addo and Marakele elephants. The table shows the gesture types that, when ordered in frequency of occurrence, represent 70% of observed cases in the two elephant populations. Gesture types shared between the two populations are highlighted in bold

Population	Gesture type	Frequency	Total	Percentage	Cumulative percentage
Addo	**Ear Spread**	108	616	17.53	17.53
	Trunk Fling	80	616	12.99	30.52
	**Ear Flap**	75	616	12.18	42.70
	Trunk Touch recipient	41	616	6.66	49.36
	**Trunk Push recipient**	39	616	6.33	55.69
	Trunk Over self	30	616	4.87	60.56
	Trunk Swing	21	616	3.41	63.97
	Trunk Reach	20	616	3.25	67.22
	Ear Slide	12	616	1.95	69.17
Marakele	**Ear Flap**	52	221	23.53	23.53
	**Ear Spread**	29	221	13.12	36.65
	**Trunk Push recipient**	17	221	7.69	44.34
	Trunk Splash	16	221	7.24	51.58
	Head Shake	10	221	4.52	56.10
	Body Turn at	7	221	3.17	59.27
	Ear Fold	7	221	3.17	62.44

	Head Rest on recipient	6	221	2.71	65.15
	Rump Push recipient	6	221	2.71	67.86

Supplementary references

Eleuteri, V., Bates, L. A., Rendle-Worthington, J., Hobaiter, C., & Stoeger, A. (2024). Multimodal communication and audience directedness in the greeting behaviour of semi-captive African savannah elephants. *Communications Biology*, *7*(1), 1–12. https://doi.org/10.1038/s42003-024-06133-5

Fowler, M., & Mikota, S. K. (2008). *Biology, Medicine, and Surgery of Elephants*. John Wiley & Sons.

Schel, A. M., Bono, A., Aychet, J., Pika, S., & Lemasson, A. (2022). Intentional gestural communication amongst red-capped mangabeys (Cercocebus torquatus). *Animal Cognition*. https://doi.org/10.1007/s10071-022-01615-7
